# Loss of *HAT1* expression confers BRAFV600E inhibitor resistance to melanoma cells by activating MAPK signaling via IGF1R

**DOI:** 10.1038/s41389-020-0228-x

**Published:** 2020-05-05

**Authors:** Suresh Bugide, Keshab Raj Parajuli, Suresh Chava, Rudradip Pattanayak, Deborah L. Della Manna, Deepmala Shrestha, Eddy S. Yang, Guoping Cai, Douglas B. Johnson, Romi Gupta

**Affiliations:** 1 Department of Biochemistry and Molecular Genetics, University of Alabama at Birmingham, Birmingham, AL 35322 USA; 2 Department of Radiation Oncology, O’Neal Comprehensive Cancer Center, University of Alabama at Birmingham, Birmingham, AL 35322 USA; 3 Department of Pathology, Yale University School of Medicine, New Haven, CT 06510 USA; 4 Department of Medicine, Vanderbilt University Medical Center, Nashville, TN 37240 USA

**Keywords:** Melanoma, Cancer genetics

## Abstract

BRAF inhibitors (BRAFi) have been approved for the clinical treatment of *BRAF*-mutant metastatic melanoma. Although initial responses to BRAFi are generally favorable, acquired BRAFi resistance emerges rapidly, resulting in treatment failure. Only some of the underlying mechanisms responsible for BRAFi resistance are currently understood. Here, we showed that the genetic inhibition of histone acetyltransferase 1 (*HAT1*) in *BRAF*-mutant melanoma cells resulted in BRAFi resistance. Using quantitative immunofluorescence analysis of patient sample pairs, consisting of pre-treatment along with matched progressed BRAFi + MEKi-treated melanoma samples, HAT1 downregulation was observed in 7/11 progressed samples (~63%) in comparison with pre-treated samples. Employing NanoString-based nCounter PanCancer Pathway Panel-based gene expression analysis, we identified increased MAPK, Ras, transforming growth factor (TGF)-β, and Wnt pathway activation in HAT1 expression inhibited cells. We further found that MAPK pathway activation following the loss of *HAT1* expression was partially driven by increased insulin growth factor 1 receptor (IGF1R) signaling. We showed that both MAPK and IGF1R pathway inhibition, using the ERK inhibitor SCH772984 and the IGF1R inhibitor BMS-754807, respectively, restored BRAFi sensitivity in melanoma cells lacking *HAT1*. Collectively, we show that the loss of *HAT1* expression confers acquired BRAFi resistance by activating the MAPK signaling pathway via IGF1R.

## Introduction

Melanoma is the deadliest form of skin cancer, accounting for ~80% of cancer-related deaths^1^. Both genetic and nongenetic factors contribute to melanoma initiation and progression, and exposure to UV radiation is the leading cause of nongenetic melanoma development^2,3^. Mutations in a number of genes have been shown to be involved in the initiation and progression of melanoma^4,5^. In particular, large-scale genomic DNA sequencing has identified activating *BRAF* mutations in ~50% of all melanoma cases^6,7^. In addition, mutations in *NRAS*, neurofibromin 1 (*NF1*), cyclin-dependent kinase inhibitor 2A (*CDKN2A*), microphthalmia-associated transcription factor (*MITF*), and phosphatase and tensin homolog (*PTEN*) have been shown to play important roles in melanomagenesis^8,9^.

BRAF is a member of the Raf kinase family, and oncogenic mutations in *BRAF* activate the mitogen-activating protein kinase (MAPK)/extracellular signal-related kinase (ERK) kinase (MEK)→ERK signaling pathway, which is required for melanoma growth and metastasis^10–12^. More than 30 different mutations have been reported in *BRAF*, associated with melanoma and other cancers^7,13^. The V600E mutation in *BRAF* has been identified in 90% of cases, followed by the V600K mutation, which has been found in 5% of cases, whereas other mutations, such as V600R, V600E2, and V600D, are found at even lower frequencies^7,14^.

Because over 50% of melanoma patients harbor oncogenic mutations in *BRAF* and *BRAF*-mutant melanoma cells depend upon *BRAF* mutations for the growth and survival, several BRAF inhibitors (BRAFi) have been approved by the US Food and Drug Administration (US FDA), including vemurafenib and dabrafenib, for the clinical treatment of metastatic melanoma^15,16^. Although, BRAFi produce impressive initial clinical responses against *BRAF*-mutant metastatic melanoma^17,18^, the durability of the BRAFi response is limited by the rapid emergence of acquired BRAFi resistance, often within a few months of treatment initiation^19^. Therefore, studies have been conducted to discern the mechanisms underlying acquired BRAFi resistance, resulting in the identification of several mechanisms associated with BRAFi resistance^20–22^. However, a subset of melanoma-resistance mechanisms that are responsible for acquired BRAFi resistance remain unknown^23^.

In order to determine the potential roles played by epigenetic regulators during the development of BRAFi resistance in melanoma, we had performed a large-scale, unbiased epigenome-wide short-hairpin RNA (shRNA) screen, targeting 363 epigenetic regulators^24^. This screen led to the identification of histone acetyltransferase 1 (*HAT1*) as one of candidate genes that mediates BRAFi resistance. In this study, we focused on determining the role of *HAT1* in acquired BRAFi resistance and understanding the mechanism behind HAT1 loss-induced acquired BRAFi resistance. We also performed experiments to establish the clinical relevance of *HAT1* during the development of BRAFi resistance in melanoma patients.

Our results showed that the loss of *HAT1* expression resulted in the development of BRAFi resistance, in part due to the activation of the MAPK pathway by insulin growth factor 1 receptor (IGF1R). Using patient-derived melanoma samples, we found that a large majority of progressed samples (63%), from patients treated with BRAFi or BRAFi + MEK inhibitor (MEKi), showed reduced *HAT1* expression levels compared with matched pre-treatment melanoma samples, indicating that *HAT1* is clinically relevant. Thus our study indicates that loss of HAT1 is one of the crucial mechanism that drives BRAFi resistance in melanoma.

## Results

### Loss of histone acetyltransferase 1 (HAT1) expression confers acquired resistance to BRAFi

Epigenetic gene regulation mechanisms play important roles in key aspects of tumor growth and metastasis and are associated with the development of drug resistance^25–27^. Therefore, to determine the potential roles played by epigenetic regulators during the development of BRAFi resistance, we previously performed a large-scale, unbiased epigenome-wide, shRNA screen that targeted 395 known and predicted epigenetic regulators using 1875 shRNAs^24^. This shRNA screen resulted in the identification of *HAT1*, but the role of *HAT1* during acquired BRAFi resistance was not characterized.

**Fig. 1:**
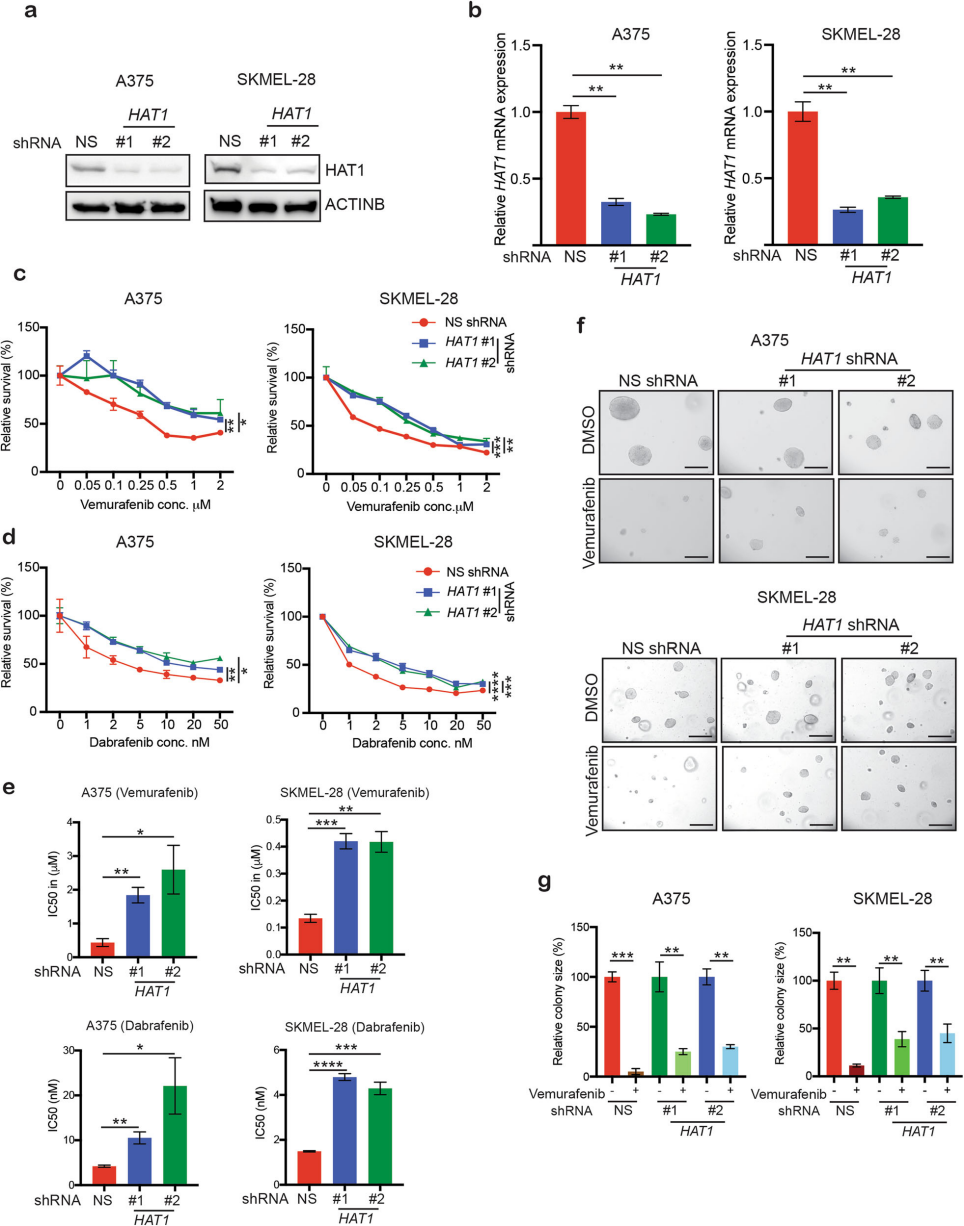
Loss of *HAT1* expression confers resistance to BRAF inhibitors. **a** Indicated melanoma cell lines, expressing either nonspecific (NS) shRNA or *HAT1* shRNAs, were analyzed for HAT1 protein expression levels by immunoblotting. ACTINB was used as a loading control. **b** Indicated melanoma cell lines, expressing either a nonspecific (NS) shRNA or *HAT1* shRNAs, were analyzed by qRT-PCR. Actin was used as an internal control**. c** Relative survival rates of A375 and SKMEL-28 cells, expressing either nonspecific (NS) shRNA or the indicated *HAT1* shRNA, upon treatment with vemurafenib for 3 days, as measured by MTT assay. **d** Relative survival rates of A375 and SKMEL-28 cells, expressing either nonspecific (NS) shRNA or the indicated *HAT1* shRNA, upon treatment with dabrafenib for 3 days, were measured by MTT assay. **e** IC50 values for the MTT data presented in panels **c** and **d**. **f** A375 or SKMEL-28 cells, expressing either a nonspecific (NS) shRNA or *HAT1* shRNAs, were treated with DMSO or vemurafenib (1 μM) and analyzed by the soft-agar assay. Scale bar is 100 µm. **g** Relative colony sizes for the data presented in panel **f**. Data are presented as the mean ± SEM, *p < 0.05, **p < 0.01, ***p < 0.001, ****p < 0.000, calculated using Student’s *t* test.

In this study, we examined whether the loss of *HAT1* expression confers BRAFi resistance in melanoma cells. We first knocked down *HAT1* expression, using shRNAs in *BRAF*-mutant melanoma cell lines (A375 and SKMEL-28) (Fig. 1a, b). As a control, a nonspecific (NS) shRNA was used.

Melanoma cells expressing either NS or *HAT1* shRNAs (*HAT1* knockdown) were then tested for their sensitivity to the BRAFi vemurafenib, in a 3-(4,5-dimethylthiazol-2-yl)-2,5-diphenyl tetrazolium bromide (MTT)-based short-term cell-survival assay. Our results showed that the knockdown of *HAT1* conferred resistance to vemurafenib (Fig. 1c, e). Next, we determined whether these findings were specific to vemurafenib or could be extended to another BRAFi, dabrafenib. Dabrafenib is a more selective, reversible, ATP-competitive kinase inhibitor that inhibits BRAFV600E and is currently approved for the clinical treatment of melanoma patients^18,28^. We found that the knockdown of *HAT1* also conferred resistance to dabrafenib (Fig. 1d, e).

To further fortify our results, we performed a soft-agar assay to determine the anchorage-independent growth abilities of *HAT1*-knockdown melanoma cells in the presence of vemurafenib. Our results showed that *HAT1*-knockdown *BRAF*-mutant melanoma cells were resistant to vemurafenib treatment compared with cells expressing a control NS shRNA (Fig. 1f, g). Taken together, these results demonstrated that the loss of *HAT1* expression resulted in acquired BRAFi resistance in melanoma cells.

### *HAT1* knockout in melanoma cells confers resistance to BRAFi in a long-term survival assay

**Fig. 2:**
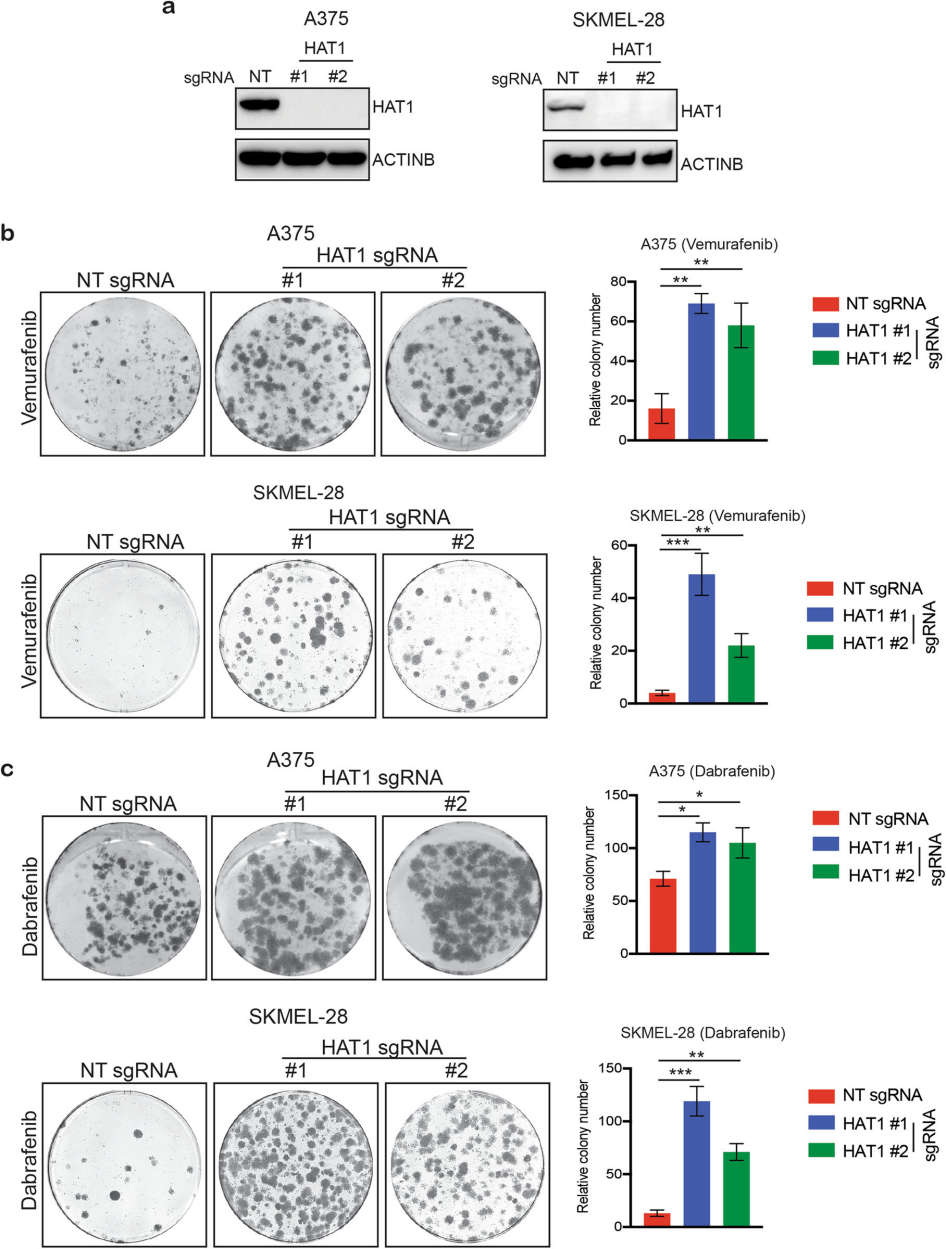
*HAT1*-knockout-mediated resistance leads to the long-term survival of melanoma cells treated with BRAF inhibitor. **a** Immunoblotting for the indicated proteins in A375 and SKMEL-28 *HAT1*-knockout (*HAT1-*KO) cells. **b** Clonogenic assay results for A375 and SKMEL-28 cells, expressing non-targeting (NT) or *HAT1* sgRNAs, in the presence of vemurafenib (3 μM) (left). Colony number for the data are shown (right). **c** Clonogenic assay results for A375 and SKMEL-28 cells, expressing NT or *HAT1* sgRNAs, in the presence of dabrafenib (100 nM) (left). Colony number for the data presented (right). Data are presented as the mean ± SEM, *p < 0.05, **p < 0.01, ***p < 0.001, calculated using Student’s *t* test.

To emulate the clinical scenario, we performed a clonogenic survival assay to examine the long-term effects of reduced *HAT1* expression on the development of BRAFi resistance. As shown in Supplementary Fig. S1, we found that *HAT1*-knockdown cells did not show BRAFi resistance in the long-term survival assay, suggesting that the partial reduction of HAT1 protein levels may not be sufficient to cause BRAFi resistance in the long-term. Because a partial reduction in HAT1 protein levels, following the shRNA-mediated *HAT1* knockdown in melanoma cells, was unable to cause BRAFi resistance to vemurafenib in a clonogenic long-term survival assay, we generated a complete *HAT1* knockout (*HAT1-*KO) in *BRAF*-mutant melanoma cells using a clustered regularly interspaced short palindromic repeats (CRISPR)/CRISPR associated (Cas)9-based approach^29,30^. We observed that *HAT1*-KO melanoma cells showed complete loss of HAT1 protein expression (Fig. 2a). Using *HAT1*-KO cells, we performed a long-term survival assay and found that these melanoma cells showed resistance to both vemurafenib and dabrafenib (Fig. 2b, c). These results confirmed that the complete loss of *HAT1* expression resulted in BRAFi resistance in the long-term.

### HAT1 protein is downregulated in patient-derived melanoma samples after BRAFi and BRAFi + MEKi treatment

**Fig. 3:**
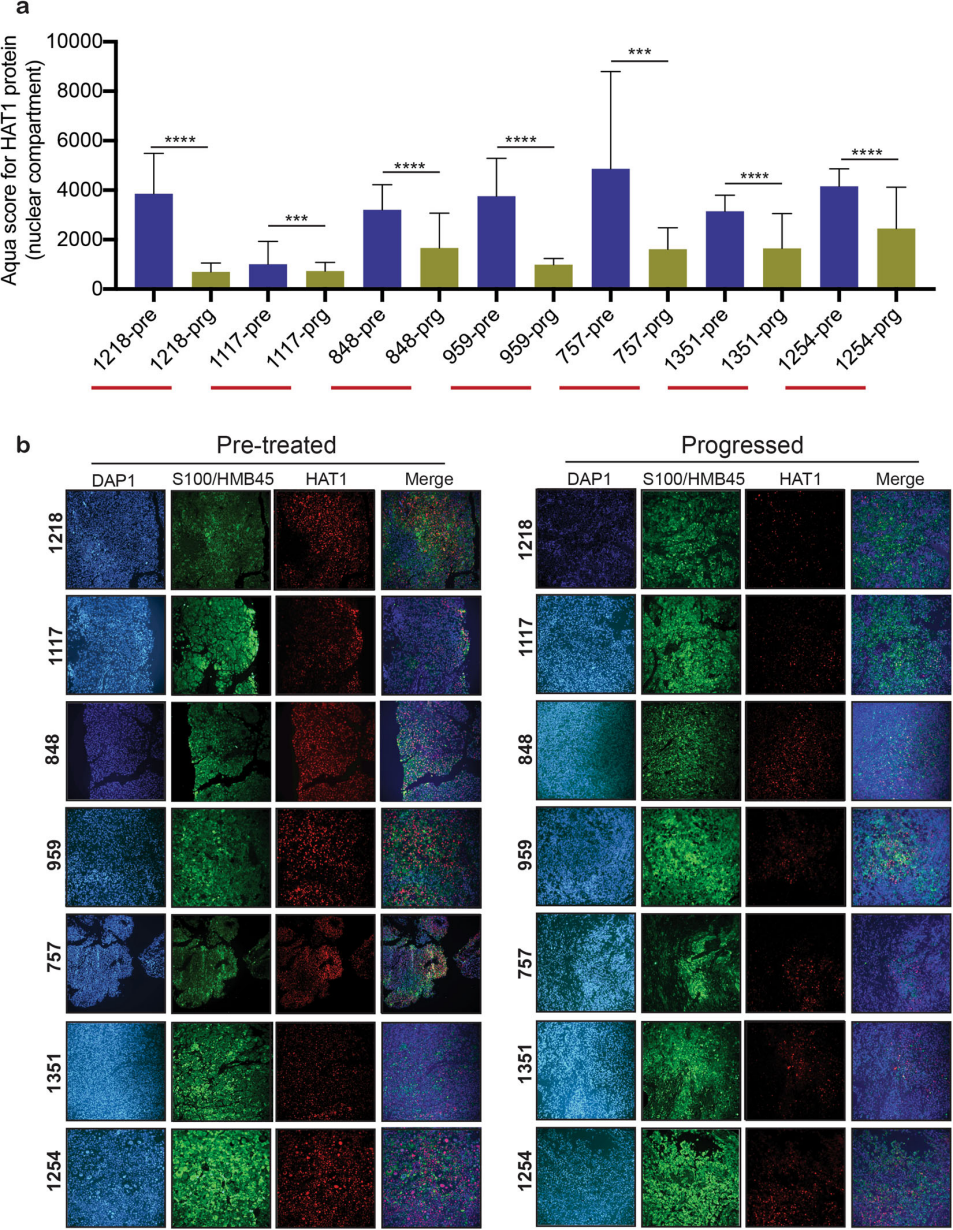
HAT1 protein is downregulated in progressed patient-derived melanoma samples following BRAF inhibitor treatment. **a** Average AQUA scores for pre-treatment and progressed melanoma samples from patients treated with BRAFi or BRAFi + MEKi. **b** Representative AQUA immunofluorescence scores and merged images of the indicated matched pre-treatment and progressed melanoma samples, stained for DAPI, S100/HMB45, and HAT1. Data are presented as the mean ± SEM. ***p < 0.001, ****p < 0.0001, calculated using Student’s *t* test.

The clonogenic long-term survival assay mimics the clinical circumstances, in which patients are treated for relatively long periods of time with a given drug. Because *HAT1*-KO melanoma cells became BRAFi resistant during a clonogenic long-term survival assay, we next tested whether *HAT1* expression was downregulated in clinical samples taken from patients who experienced disease progression following treatment with BRAFi or BRAFi+MEKi. We analyzed 11 sets of matched, patient-derived, melanoma samples, consisting of a pre-treatment sample and a progressed sample, taken after treatment with either BRAFi or BRAFi+MEKi (Supplementary Table 1). To quantitatively monitor changes in HAT1 protein levels, we performed an automated quantitative analysis (AQUA)-based immunofluorescence analysis. Our results showed that 7 out of 11 cases of progressed melanoma samples, from patients previously treated with BRAFi or BRAFi+MEKi, showed lower expression levels of HAT1 protein compared with their respective, matched, pre-treatment samples (Fig. 3a, b; Supplementary Fig. S2 and Supplementary Table 1). These results further established that BRAFi resistance following the loss of *HAT1* expression represents a clinically relevant mechanism among BRAFi- and BRAFi + MEKi-treated melanoma patients.

### The loss of *HAT1* expression leads to the activation of multiple oncogenic signaling pathways in *BRAF*-mutant melanoma cells

**Fig. 4:**
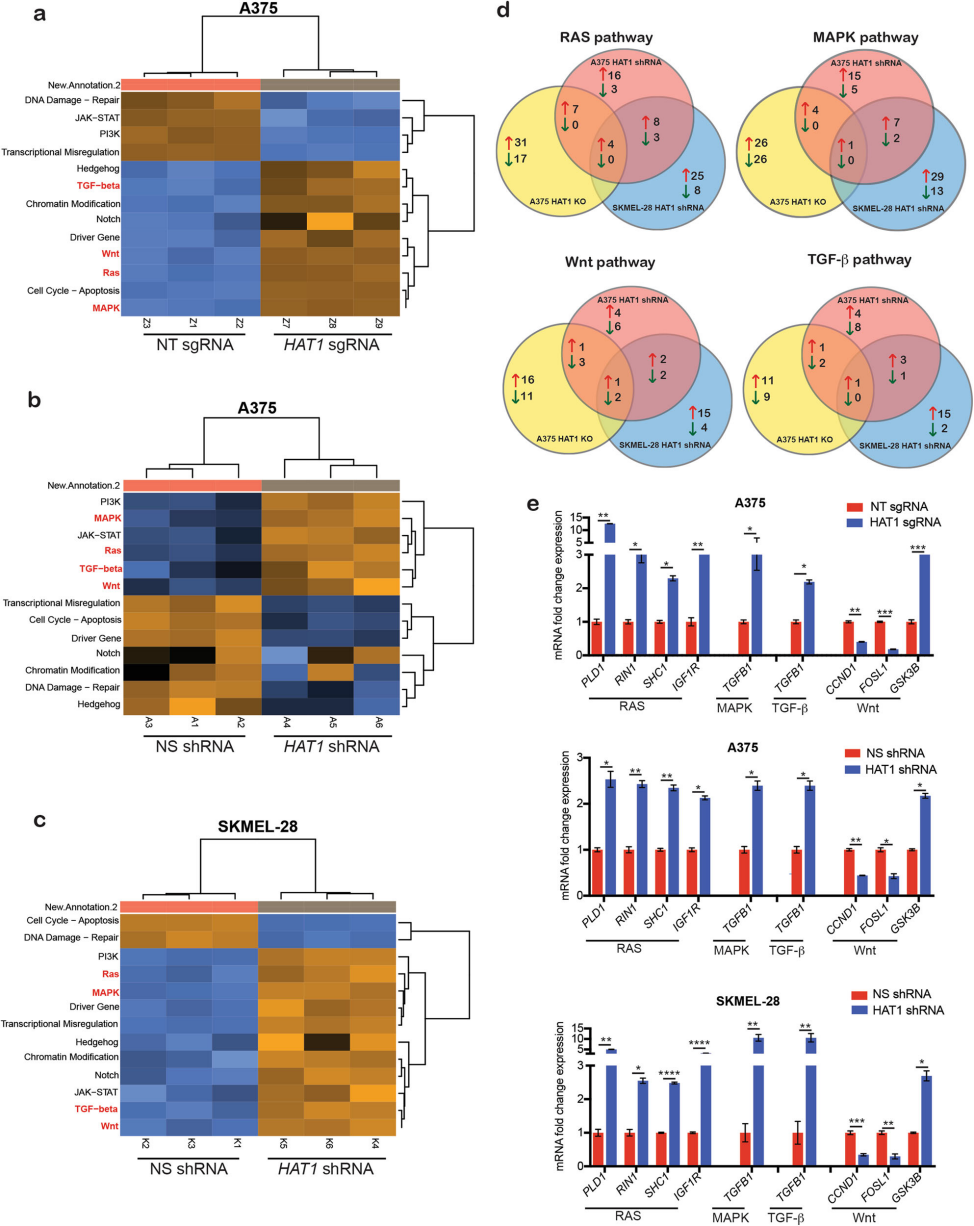
The loss of *HAT1* expression leads to the activation of alternative signaling pathways, which contribute to BRAFi resistance in melanoma cells. **a** Heatmap for A375 cells, expressing non-targeting (NT) or *HAT1* sgRNAs. **b** Heatmap for A375 cells, expressing nonspecific (NS) or *HAT1* shRNAs. **c**. Heatmap for SKMEL-28 cells, expressing NS or *HAT1* shRNAs. **d** Venn diagram showing the genes that were significantly up- and downregulated in A375 *HAT1* knockout (KO), A375 *HAT1* knockdown (KD), and SKMEL-28 HAT1 KD cells in the Ras, MAPK, Wnt, and TGF-β signaling pathways. **e** Fold changes of the genes associated with the Ras, MAPK, TGF-β, and Wnt signaling pathways that were significantly altered in A375 cells expressing NT or *HAT1* sgRNAs, A375 cells expressing NS or *HAT1* shRNAs, and SKMEL-28 cells expressing NS or *HAT1* shRNAs. Data are presented as the mean ± SEM, *p < 0.05, **p < 0.01, ***p < 0.001, calculated using Student’s *t* test.

HAT1 is a histone acetyltransferase that regulates gene expression^31,32^. Therefore, we speculated that changes in gene expression may underlie the development of BRAFi resistance following reduced *HAT1* expression. To measure changes in gene expression, we performed a NanoString-based gene expression analysis, using the nCounter PanCancer Pathway Panel for Gene Expression. The nCounter PanCancer Pathway Panel monitors the expression of over 700 genes, spread across 13 canonical cancer hallmark pathways (Supplementary Fig. S3). These includes genes in chromatin modification, Hedgehog, Wnt, Notch, apoptosis, cell cycle, RAS, PI3K, STAT, MAPK, TGF-β, DNA damage control, transcriptional regulation that drive different aspect of cancer growth, progression, and treatment response. Our results showed that common pathways that were upregulated in both *HAT1*-knockdown and *HAT1*-KO melanoma cells, includes TGF-β, Wnt, Ras, and MAPK pathways (Fig. 4a–c; Supplementary Table 2). We then analyzed the expression levels of genes associated with the TGF-β, Wnt, Ras, and MAPK signaling pathways and found the following changes in both *HAT1*-knockdown and *HAT1*-KO melanoma cells: in the Ras pathway, phospholipase D1 (*PLD1)*, Ras and Rab interactor 1 (*RIN1*), insulin-like growth factor 1 receptor (*IGF1R*), and Src homology 2 domain-containing (SHC) transforming protein 1 (*SHC1)* were significantly upregulated; for both the TGF-β and MAPK signaling pathways, *TGFβ1* was significantly upregulated; and in the Wnt signaling pathway, glycogen synthase kinase 3 β (*GSK3B*) was significantly upregulated, whereas cyclin D1 (*CCND1*) and Fos-related antigen 1 (*FOSL1*) were significantly downregulated, (Fig. 4d, e; Supplementary Table 2).

In *HAT1*-KO melanoma cells, genes associated with other signaling pathways were also upregulated, including Hedgehog, notch, cell cycle-apoptosis, chromatin modification, and driver genes (Fig. 4a). These upregulated pathways potentially contribute to the increased resistance phenotype during long-term survival assays observed for *HAT1-*KO cells compared with that observed for *HAT1*-knockdown cells. Overall, our results demonstrated that the loss of *HAT1* expression resulted in the upregulation of both MAPK-independent and MAPK-dependent pathways, leading to the acquisition of BRAFi resistance in melanoma cells.

### The activation of IGF1R following the loss of *HAT1* expression results in the activation of MAPK pathways in *BRAF*-mutant melanoma cells

**Fig. 5:**
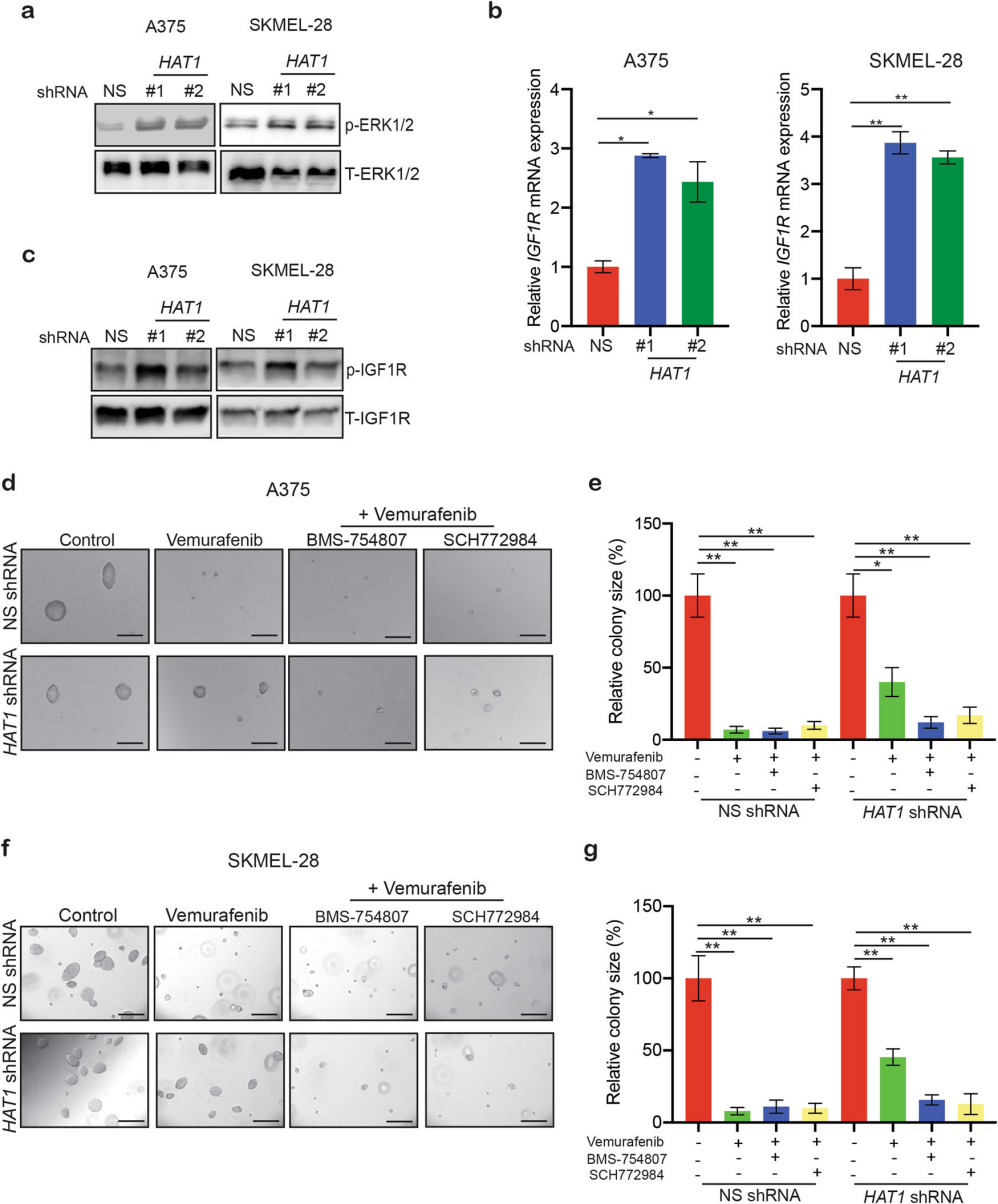
Loss of *HAT1* expression leads to the IGF1R-dependent activation of the MAPK signaling pathway, which confers BRAFi resistance in melanoma cells. **a** Immunoblotting for phospho- and the total ERK in A375 and SKMEL-28 cells expressing nonspecific NS or *HAT1* shRNAs. **b** Relative IGF1R mRNA levels in A375 and SKMEL-28 cells expressing nonspecific NS or *HAT1* shRNAs were analyzed by qRT-PCR. Actin was used as an internal control. **c** Immunoblotting for phospho- and the total IGF1R in A375 and SKMEL-28 cells expressing nonspecific NS or *HAT1* shRNAs. **d**, **f** A375 or SKMEL-28 cells expressing a nonspecific (NS) shRNA or *HAT1* shRNAs were treated with DMSO, BRAF inhibitor vemurafenib (1 μM), IGF1R inhibitor, BMS-754807 (0.1 μM) and ERK inhibitor, SCH772984 (0.2 μM), which was analyzed by soft-agar assay. **e**, **g** Relative colony size for data presented in panels **d** and **f**. Data are presented as the mean ± SEM, *p < 0.05, **p < 0.01, calculated using Student’s *t* test.

**Fig. 6:**
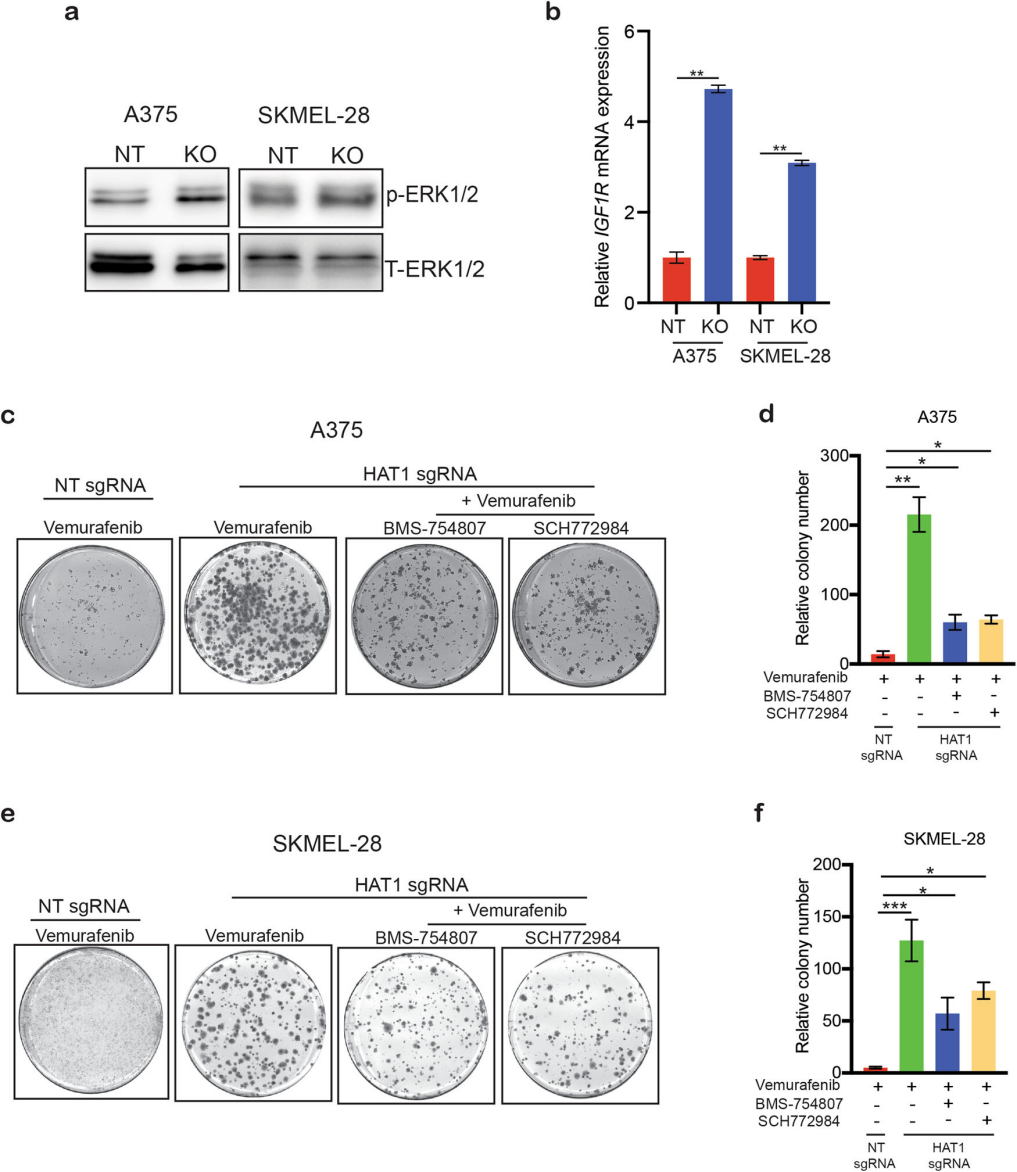
Pharmacological inhibition of IGF1R and ERK suppresses the growth of BRAFi-resistant cells following the loss of *HAT1* expression. **a** Immunoblotting for phospho- and total ERK in A375 cells expressing non-targeting NT or *HAT1* sgRNAs. **b** Relative IGF1R mRNA levels in A375 cells expressing non-targeting NT or *HAT1* sgRNAs was analyzed by qRT-PCR. Actin was used as internal control. **c**, **e** A375 or SKMEL-28 cells expressing a non-targeting (NT) sgRNA or *HAT1* sgRNAs were treated with DMSO, BRAF inhibitor vemurafenib (3 μM), IGF1R inhibitor, BMS-754807 (1 μM) and ERK inhibitor, SCH772984 (1 μM) was analyzed by clonogenic assay. **d**, **f** Relative colony number for data presented in panels **c** and **e**. Data are presented as the mean ± SEM, *p < 0.05, **p < 0.01, ***p < 0.001, calculated using Student’s *t* test.

We next used immunoblotting analyses to measure the protein expression levels of the various signaling pathways identified during the NanoString analysis. We determined the levels of the following proteins: phospho- and total ERK1/2, to assess the MAPK signaling pathway; TGFβ1, to assess both the TGF-β and MAPK signaling pathways; and beta-catenin, to assess the Wnt signaling pathway. Our results showed that phospho-ERK1/2 protein levels were significantly upregulated in both *HAT1-*knockdown and *HAT1-*KO melanoma cells (Figs. 5a and 6a). However, we did not observe any significant changes in TGFβ1 or beta-catenin protein levels in either *HAT1*-knockdown or *HAT1*-KO melanoma cells (Supplementary Fig. S4).

We attempted to determine the mechanism through which phospho-ERK1/2 levels were elevated following the loss of *HAT1* expression in *BRAF*-mutant melanoma cells. We first determined the levels of dual-specific phosphatases (DUSPs) in cells with reduced *HAT1* expression because some DUSPs regulate MAPK signaling by inhibiting the activation of ERK1/2^33^. Our results showed that DUSP levels were downregulated only in *HAT1-*KO melanoma cells, whereas in *HAT1-*knockdown melanoma cells, the DUSP expression levels did not change significantly (Supplementary Fig. S5).

In addition, because increased *IGF1R* expression levels were identified in the nCounter PanCancer Pathway Panel for Gene Expression, in both *HAT1*-knockdown and *HAT1-*KO *BRAF*-mutant melanoma cells (Fig. 4e), we analyzed the mRNA levels as well as protein levels of phospho- and total IGF1R in these samples. Strikingly, we found that IGF1R mRNA levels as well as phospho-IGF1R was significantly upregulated in both *HAT1*-knockdown and *HAT1-*KO cells (Figs. 5b, c and 6b). Previous studies have shown that the activation of IGF1R signaling can activate the MAPK/Ras pathway^34,35^. In our study, we found that in melanoma cells with reduced *HAT1* expression levels, IGF1R levels were upregulated, leading to the activation of MAPK/Ras signaling pathway and conferring BRAFi resistance.

### The pharmacological inhibition of IGF1R or ERK partially rescues the BRAFi-resistance phenotype in melanoma cells following the loss of *HAT1* expression

Because our results showed that *HAT1*-knockdown and *HAT1*-KO BRAFi-resistant melanoma cells demonstrated increased IGF1R expression levels and increased MAPK pathway activation, we examined whether the ERK inhibitor SCH772984^36^ or the IGF1R inhibitor BMS-754807^37^ could resensitize *HAT1*-knockdown and *HAT1*-KO BRAFi-resistant melanoma cells to BRAF inhibitor vemurafenib. We found that treatment with either the ERK or IGF1R inhibitors was able to restore sensitivity to vermurafenib in both *HAT1*-knockdown and *HAT1*-KO BRAFi-resistant melanoma cells, in both the soft-agar assay (Fig. 5d–g) and in the clonogenic long-term survival assay (Fig. 6c–f). These results confirmed the loss of *HAT1* expression resulted in the activation of the MAPK pathway by IGF1R, which conferred BRAFi resistance; therefore, IGF1R or ERK1/2 inhibitors represent pharmacologically tractable options for inhibiting the growth of BRAFi-resistant melanoma cells.

## Discussion

Oncogenic mutations in *BRAF* have been identified in ~50% of melanoma cases. BRAFi, either alone or in combination with MEKi, represents therapeutic options for the treatment of *BRAF*-mutant metastatic melanomas. However, due to the rapid emergence of acquired BRAFi resistance, the clinical benefits of these therapies are often limited^22,38,39^.

Previous studies have identified several mechanisms associated with the development of BRAFi resistance. For example, the mutational activation of other oncogenes, such as *NRAS*, *MEK1*, and *MEK2*, the overexpression of *CRAF*, the dimerization of oncogenic BRAF, and the upregulation of the PTEN-PI3K-AKT signaling pathway have all been identified as potential mechanisms underlying the development of BRAFi/BRAFi + MEKi resistance^39–43^.

**Fig. 7:**
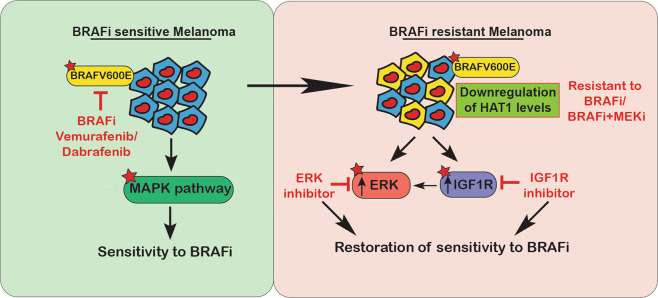
Model depicting the mechanism of HAT1 loss-induced BRAFi resistance. One of the mechanisms by which loss of *HAT1* drives acquired BRAFi resistance is via IGF1R-dependent activation of MAPK signaling pathway, and the pharmacological targeting of IGF1R or ERK1/2 restores the sensitivity of melanoma cells with reduced *HAT1* expression levels to BRAFi.

In this study, we investigated the role played by *HAT1* in the regulation of BRAFi resistance in melanoma cells and showed that the loss of *HAT1* expression contributed to the development of BRAFi resistance (Fig. 7). HAT1 is a type-B histone acetyltransferase that acetylates newly synthesized H3 and H4 histones and participates in chromatin assembly^44,45^. HAT1 has also been shown to induce apoptosis by upregulating Fas expression in lung cancer cells^46^. In addition, Ras-ERK1/2 signaling has been shown to promote the development of osteosarcoma through the regulation of H4K12Acetyl, via HAT1^47^. We showed that the loss of *HAT1* expression was associated with the development of resistance to BRAFi, such as vemurafenib and dabrafenib. We also confirmed that ~63% of progressed samples, from *BRAF*-mutant melanoma patients who experienced disease progression following BRAFi or BRAFi + MEKi therapy, showed significantly reduced HAT1 expression levels compared with matched pre-treatment samples. These results collectively demonstrated that the loss of *HAT1* expression during the acquired resistance to BRAFi and BRAFi + MEKi represents a clinically relevant event.

We also performed gene expression NanoString analysis to further understand the underlying mechanisms that result in the development of BRAFi resistance following the loss of *HAT1* expression. In this assay, we analyzed 700 genes, clustered into 13 hallmark cancer pathways. These studies identified the upregulation of the Ras, MAPK, Wnt, and TGF-β signaling pathways in melanoma cells lacking *HAT1*. We then confirmed that among these four upregulated signaling pathways, the MAPK pathway was significantly upregulated in BRAFi-resistant cells, following the loss of *HAT1* expression. We further found that cells lacking *HAT1* expression showed increased IGF1R signaling activity. IGF1R has been shown to activate the MAPK signaling pathway, promoting cell growth and proliferation, and can drive acquired resistance to various drugs^48,49^. Therefore, IGF1R signaling pathway inhibitors have been utilized as alternative strategies for the treatment of drug-resistant cancer cells and to counteract drug resistance^41,50^.

We also found that the pharmacological targeting of the MAPK signaling pathway via IGF1R and ERK1/2 inhibitors was able to partially restore the sensitivity of *HAT1*-knockdown and *HAT1*-KO melanoma cells to BRAFi. ERK1/2 is the most downstream kinase of the Ras–BRAF–MEK–ERK cascade, and targeting ERK1/2 can overcome resistance mechanisms caused by changes in upstream pathways^51^. In particular, ERK1/2 inhibitors have shown some activity in BRAFi- or BRAFi + MEKi-resistant melanomas^52,53^. Although not directly tested in this study, we predict that HAT1 expression levels in samples from patients with progressed disease states can be used as an indicator of IGF1R and ERK1/2 inhibitor sensitivity, due to the activation of the IGF1R and MAPK pathways in melanoma cells expressing low levels of *HAT1*. Although, we have not directly tested the BRAFi + MEKi combination in our cell culture experiments. However, based on the activation of IGF1R and our clinical findings that HAT1 is also downregulated in patients treated with the combination of BRAFi + MEKi, it is likely that HAT1 loss will also confer resistance to BRAFi + MEKi combination therapy. Collectively, our study showed that the loss of *HAT1* expression results in acquired BRAFi resistance, in part by increasing MAPK pathway activation via IGF1R, and indicated that treatments with IGF1R and/or ERK1/2 inhibitors can enhance BRAFi efficacy and overcome the limitations associated with BRAFi and BRAFi + MEKi treatment.

## Materials and methods

### Cell culture and inhibitors

SKMEL-28, A375, and HEK293T cells were purchased from American Type Culture Collection (ATCC) and grown as recommended. Cell lines were used only after confirming the lack of *Mycoplasma* contamination using MycoAlert Mycoplasma detection kit (Lonza), and were also routinely tested for lack of mycoplasma contamination. All cell lines were passaged for 2–4 weeks between thawing and use in the described experiments. BRAFi (vemurafenib and dabrafenib), the ERK inhibitor SCH772984, and the IGF1R inhibitor BMS-754807 were purchased from Selleckem.

### RNA preparation, cDNA preparation, quantitative PCR analysis

The total RNA was extracted with TRIzol Reagent (Invitrogen) and purified using the RNeasy Mini Kit (Qiagen). cDNA was generated using the M-MuLV First Strand cDNA Synthesis Kit (New England Biolabs), according to the manufacturer’s instructions. Quantitative reverse transcriptase (RT)-PCR was performed with gene-specific primers, using the Power SYBR-Green Master Mix (Applied Biosystems), according to the manufacturer’s instructions. Actin was used as an internal control. Primer sequences are provided in Supplementary Table 3.

### shRNA and lentivirus preparation

pLKO.1 lentiviral vector-based shRNAs, targeting specific candidate genes, and NS shRNA controls were obtained from OpenBiosystems (Dharmacon). shRNA information is provided in Supplementary Table 3. Lentivirus particles were prepared by transfecting 293T cells with either gene-specific shRNA or NS shRNA plasmids, along with the lentiviral packaging plasmids, as described in detail at https://portals.broadinstitute.org/gpp/public/resources/protocols. All lentiviral transfections were performed using Effectene (Qiagen). Stable cell lines were generated by infecting melanoma cells with lentivirus particles, followed by selection with appropriate concentrations of puromycin (0.5–1.5 μg/mL), to enrich infected cells.

### Preparation of the *HAT1* single guide RNA (sgRNA) lentivirus and generation of stable cell lines

Gene-specific lentiviral *HAT1* sgRNAs were cloned into the pLentiCRISPR v2 vector. The sgRNA sequences are provided in Supplementary Table 3. For lentivirus production, sgRNAs were transfected into 293T cells, along with the PDM2.G and psPAX2 packaging plasmids, using Effectene Transfection Reagent (Qiagen), according to the manufacturer’s instructions. After 48 h, the lentivirus-containing supernatants were harvested, filtered, and used for infections. Lentiviral sgRNA-infected melanoma cells were selected using 0.6 μg/ml puromycin.

### Antibodies and immunoblot analysis

Whole-cell protein extracts were prepared using Pierce IP lysis buffer (Thermo Fisher Scientific), containing protease inhibitor mixture (Roche) and phosphatase inhibitor mixture (Sigma-Aldrich). Protein concentrations were estimated using the Bradford assay (Bio-Rad). Proteins were separated by 10 or 12% sodium dodecyl sulfate-polyacrylamide gel electrophoresis (SDS-PAGE) and transferred onto polyvinylidene fluoride (PVDF) membranes by wet transfer. PVDF membranes were blocked with 5% nonfat dry milk or 5% bovine serum albumin (BSA), as recommended for each specific antibody, washed, and probed with primary antibodies. Membranes were washed again, followed by incubation with horseradish peroxidase (HRP)-conjugated secondary antibodies (GE Healthcare). Immunoblots were developed using SuperSignal West Pico or Femto Substrates (Pierce), as necessary. All primary and secondary antibodies used in these studies are listed in Supplementary Table 3.

### Soft-agar assay

Soft-agar assays were performed by seeding between 5 × 10^3^ and 2 × 10^4^ melanoma cells, which stably expressed the indicated shRNA or cDNA constructs, onto 0.4% low-melting-point agarose (Sigma-Aldrich), layered on top of 0.8% agarose. For drug-treatment experiments, cells were then treated with DMSO, vemurafenib (1 µM), or dabrafenib (50 nM), IGF1R inhibitor, BMS-754807 (0.1 μM) and ERK inhibitor, SCH772984 (0.2 μM), and cell culture media was changed every 3 days, adding fresh drug each time. After 3–4 weeks of incubation, colonies were stained with a 0.005% crystal violet solution and imaged using a microscope. Colony sizes were measured using ImageJ software (https://imagej.nih.gov/ij/) and plotted. This experiment was performed in triplicate.

### MTT assay

For this assay, 5 × 10^3^ cells were plated in a 100-µl volume, in 96-well plates in triplicate. After 48 h, inhibitors (i.e., vemurafenib and dabrafenib), used at a range of concentrations, were mixed in 100 µl of medium and added to the cells. After 48 h of inhibitor treatment, the cell viability was evaluated by adding 20 µl of 5 mg/ml MTT solution dissolved in 1× PBS to each well and incubating for 1 h at 37 °C. The MTT solution was removed gently, and 100 µl of DMSO was added to each well. After mixing each well by pipetting, the absorbance was measured at 590 and 630 nm. An average was calculated for both readings, and then the measurement at 630 nm was subtracted from that at 590 nm. The relative growth rate was plotted with respect to vehicle-treated control cells.

### Clonogenic assay

The clonogenic abilities of cells stably expressing control or gene-specific shRNAs were measured in untreated and vemurafenib- and dabrafenib-treated conditions. For clonogenic assay, 1 × 10^5^ cells were seeded in a six-well plate in triplicate and after 24 h they were either vehicle treated or treated with vemurafenib (3 μM), dabrafenib (100 nM), IGF1R inhibitor, BMS-754807 (1 μM) and ERK inhibitor, SCH772984 (1 μM). After 3–4 weeks of treatment, colonies were fixed with a fixing solution, containing 50% methanol and 10% acetic acid, and then stained with 0.05% Coomassie blue (Sigma-Aldrich). The relative number of colonies was calculated first by counting the number of colonies for each samples and then by plotting the average numbers of colonies counted for triplicate in the indicated shRNAs versus NS shRNA.

### Patient sample acquisition

Melanoma samples were obtained through biopsies and surgical resections, performed during the standard clinical care of melanoma patients. Excess samples not required for surgical pathology assessments were stored in the Vanderbilt University melanoma tumor repository, as formalin-fixed paraffin-embedded (FFPE) samples. All patients provided consent through an Institutional Review Board-approved protocol before tissue acquisition (Vanderbilt IRB# 030220), and samples were de-identified for the analysis (Supplementary Table 1).

### Quantitative immunofluorescence analysis

Whole-tissue sections of paired pre-treatment and progressed samples from patients treated with targeted BRAFi, as described in Supplementary Table 1, were deparaffinized at 60 °C for 30 min, incubated in xylene (twice, for 20 min each), and rehydrated with ethanol (twice, in 100% ethanol for 1 min, and then in 70% ethanol for 1 min). Antigen retrieval was performed by boiling the samples for 20 min at 97 °C in citrate buffer, pH 6.0 (PT module, Lab Vision; Thermo Scientific). Slides were blocked with 30% hydrogen peroxide in methanol and then incubated with a blocking solution, containing 0.3% BSA in Tris-buffered saline and 0.05% Tween solution (TBST), for 30 min at room temperature. Slides were then incubated overnight, with a mixture of HAT1 rabbit antibodies, and S100 and HMB45 mouse antibodies (Supplementary Table 3). The next day, slides were washed and treated with Alexa 546-conjugated goat anti-mouse secondary antibody (Invitrogen), diluted 1:100 in rabbit Envision reagent (K4003, Dako), and incubated for 60 min at room temperature. For target detection, slides were treated with cyanine 5, directly conjugated to tyramide (FP1117; Perkin-Elmer), at a 1:50 dilution, for 10 min. ProLong gold mounting medium (Invitrogen), containing 4′,6-diamidino-2-phenylindole (DAPI), was used to stain nuclei. Control slides were processed for reproducibility alongside each experimental slide-staining run. Quantitative measurements of HAT1 and immunofluorescence analysis were performed using the AQUA method^54^. A tumor mask was created by binarizing the HAT1, S100, and HMB45 signals. Quantitative immunofluorescence scores were calculated by dividing the target pixel intensity by the area of the S100 and HMB45 compartments. All patient samples were scored using AQUA software, and in each sample 40–70 different spots were analyzed; the average tumor mask-normalized scores are shown in the figures.

### NanoString analysis

RNA was analyzed using the NanoString nCounter platform (Seattle, WA), through the UAB NanoString Laboratory (http://www.uab.edu/medicine/radonc/en/nanostring). All RNA samples had A260/A280 and A260/A230 ratios between 1.8 and 2.3, as recommended by the manufacturer and determined using a DeNovix DS-11 spectrophotometer (Wilmington, DE). Briefly, 100 ng of each sample was hybridized for 18 h, using Reporter and Capture Probes specific to the human PanCancer Pathways panel, and processed on the NanoString nCounter Flex system, according to the manufacturer’s instructions. This premade panel contains 730 genes involved in 13 hallmark cancer pathways (apoptosis, cell cycle, chromatin modification, DNA damage control, Hedgehog, MAPK, Notch, P13K, Ras, STAT, TGF-β, transcriptional regulation, and Wnt) as well as housekeeping genes and negative and positive controls. The samples were read at the standard 280 FOV count, the resultant RCC data files were imported into NanoString nSolver 4.0, and the raw data was used to run through the advanced analysis module. This module selects the best housekeeping genes to use during the analysis, through the GNORM program, and those selected genes were used to normalize the data.

### Statistical analysis

All experiments were conducted using at least three biological replicates. The sample size was determined based on previous experience for each experiments to detect specific effects and it was not predetermined with any statistical methods. Results for individual experiments are expressed as the mean ± standard error of the mean (SEM). For measurements of MTT assays, statistical analyses were performed by analyzing the area under the curve, using GraphPad Prism software, version 7.0, for Macintosh (GraphPad Software; https://www.graphpad.com). For the remaining experiments, *P-*values were calculated using a two-tailed, unpaired, Student’s *t* test, in GraphPad Prism software, version 7.0, for Macintosh.

## Supplementary information

Supplementary Figure Legends

Supplementary Figure 1

Supplementary Figure 2

Supplementary Figure 3

Supplementary Figure 4

Supplementary Figure 5

Supplementary Table 1

Supplementary Table 2

Supplementary Table 3
