# Supplementary material for: Loss of HAT1 expression confers BRAFV600E inhibitor resistance to melanoma cells by activating MAPK signaling via IGF1R
Source: Oncogenesis. 2020 May 5;9(5):44. doi: 10.1038/s41389-020-0228-x (PMC7200761; doi:10.1038/s41389-020-0228-x)
Supplement: Supplementary file 1 — Supplementary Figure Legends [file 41389_2020_228_MOESM1_ESM.docx]

**Supplementary Figure Legends**

**Fig. S1. Complete loss of *HAT1* expression is required for BRAFi resistance in melanoma cells**

**a.** Clonogenic assay for A375 cells, expressing non-specific (NS) or *HAT1* shRNAs, in the presence of vemurafenib (3 μM) (left). Colony numbers for the presented data (right). **b.** Clonogenic assay for A375 cells, expressing NS or *HAT1* shRNAs, in the presence of dabrafenib (100 nM) (left). Colony numbers for the presented data (right). Data are presented as the mean ± SEM, * represents p < 0.05, ns = not significant p-value, calculated using student’s t-test.

**Fig. S2. HAT1 protein levels in patient-derived, progressed melanoma samples, following BRAF inhibitor treatment**

Average AQUA scores for pre-treatment and progressed melanoma samples from patients treated with BRAFi or BRAFi+MEKi. Data are presented as the mean ± SEM, * represents p < 0.05, ns = not significant p-value, calculated using student’s t-test.

**Fig. S3. NanoString-based gene expression analysis**

NanoString-based gene expression analysis utilizes then nCounter PanCancer Pathway Panel to monitor the expression of over 730 genes, spread across 13 canonical cancer hallmark pathways.

**Fig. S4. Measurement other signaling pathway activation following the loss of *HAT1* expression**

**a.** Immunoblotting for the shown proteins in A375 cells, expressing non-targeting (NT) or *HAT1* sgRNAs. **b**. Immunoblotting for the shown proteins in A375 and SKMEL-28 cells, expressing non-specific (NS) or *HAT1* shRNAs.

**Fig. S5. Measurement of dual specific phosphatases (DUSPs) following the loss of *HAT1* expression**

**a.** mRNA expression levels for DUSPs were measured by qRT-PCR in A375 cells, expressing non-targeting (NT) or *HAT1* sgRNA, and in A375 and SKMEL-28 cells, expressing non-specific (NS) or *HAT1* shRNAs. Data are presented as the mean ± SEM, * p<0.05, ** p<0.01, *** p<0.001, **** p<0.0001, calculated using student’s t-test.
