# Supplementary figures and images for: Loss of HAT1 expression confers BRAFV600E inhibitor resistance to melanoma cells by activating MAPK signaling via IGF1R
Source: Oncogenesis. 2020 May 5;9(5):44. doi: 10.1038/s41389-020-0228-x (PMC7200761; doi:10.1038/s41389-020-0228-x)

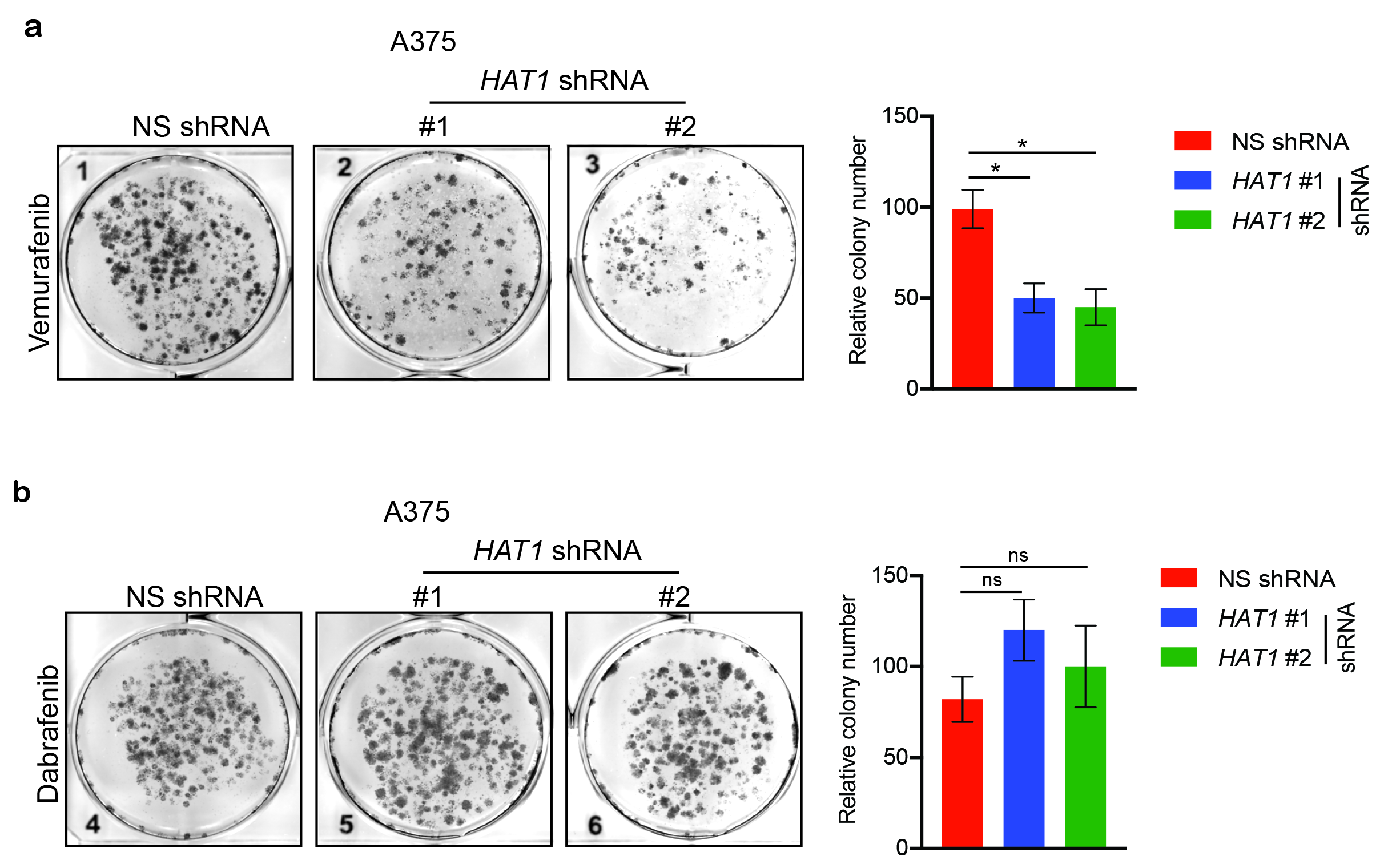

Supplement: Supplementary file 2 — Supplementary Figure 1 [file 41389_2020_228_MOESM2_ESM.tif]

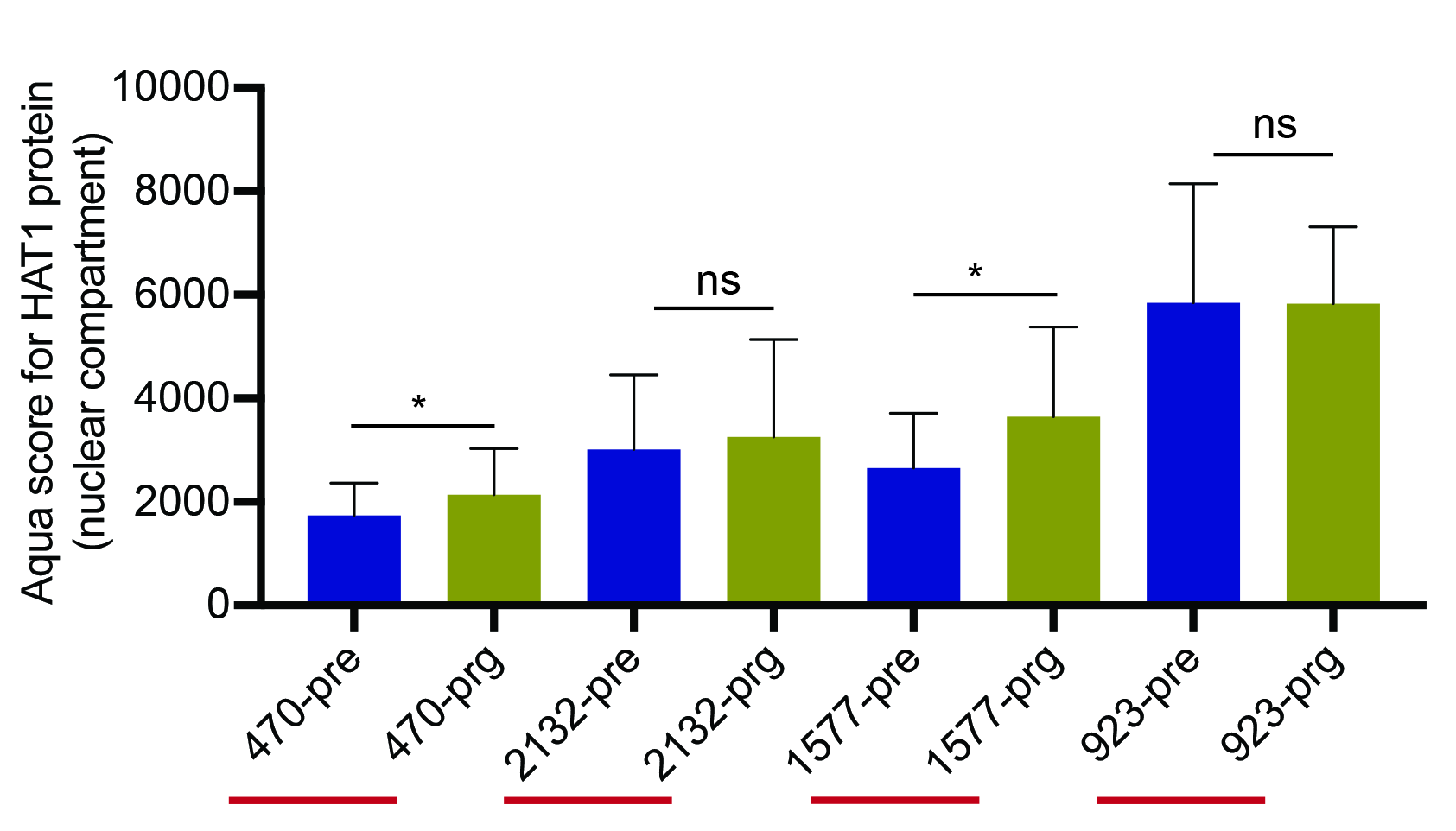

Supplement: Supplementary file 3 — Supplementary Figure 2 [file 41389_2020_228_MOESM3_ESM.tif]

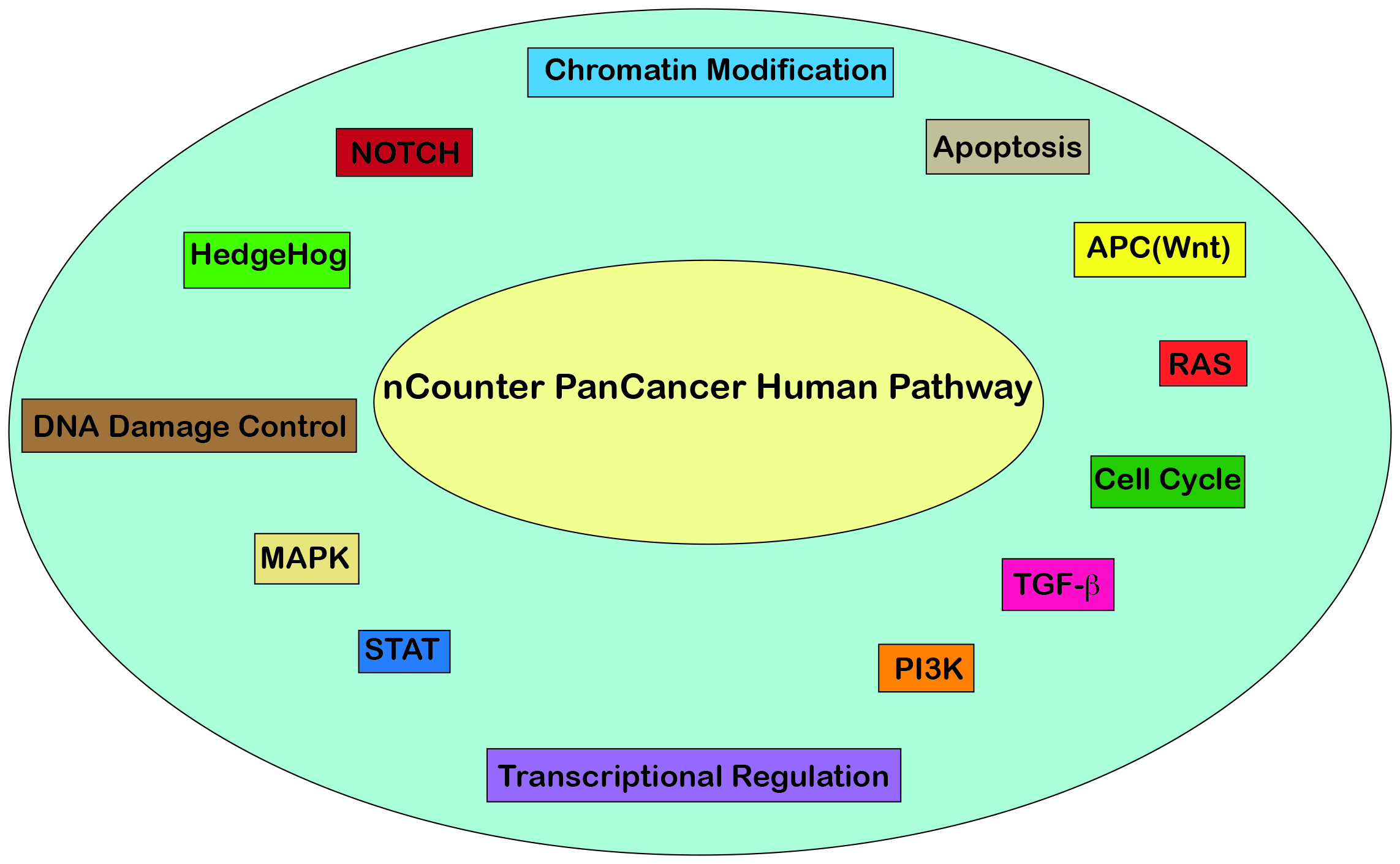

Supplement: Supplementary file 4 — Supplementary Figure 3 [file 41389_2020_228_MOESM4_ESM.tif]

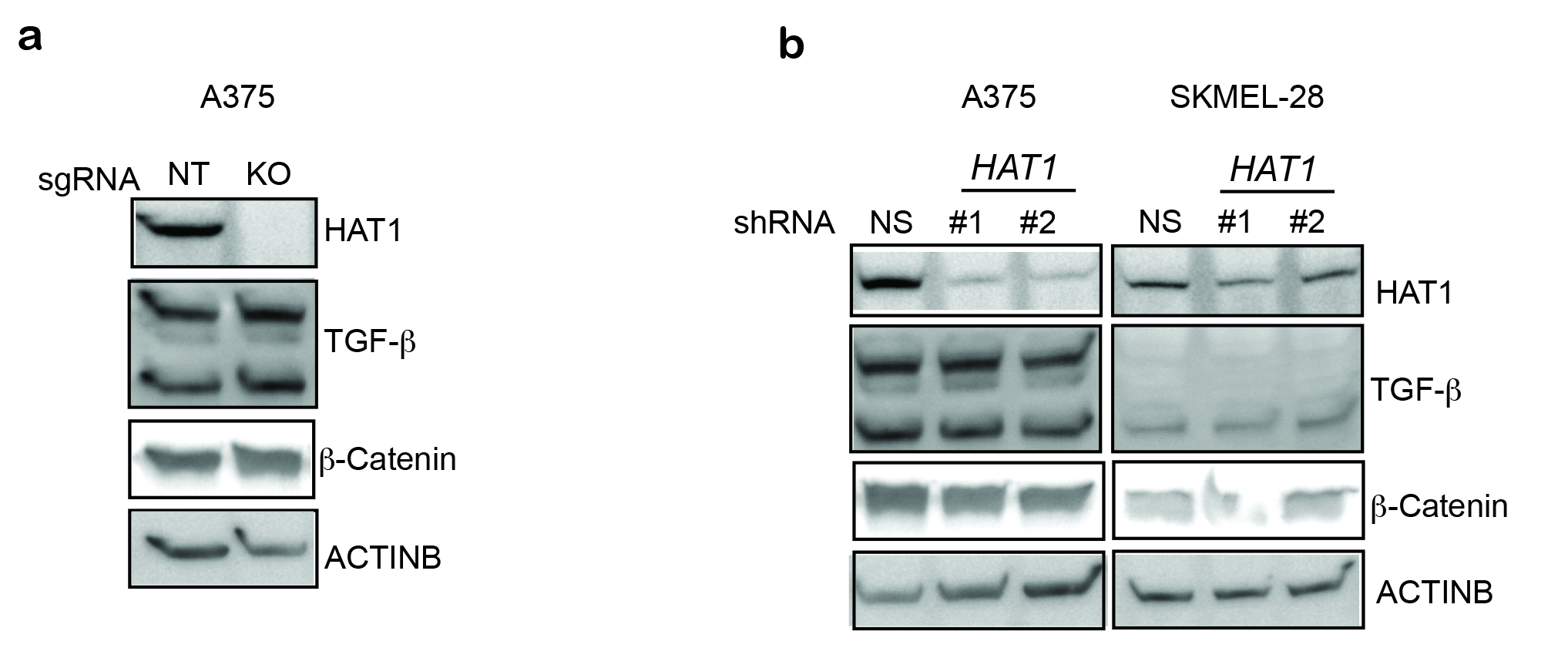

Supplement: Supplementary file 5 — Supplementary Figure 4 [file 41389_2020_228_MOESM5_ESM.tif]

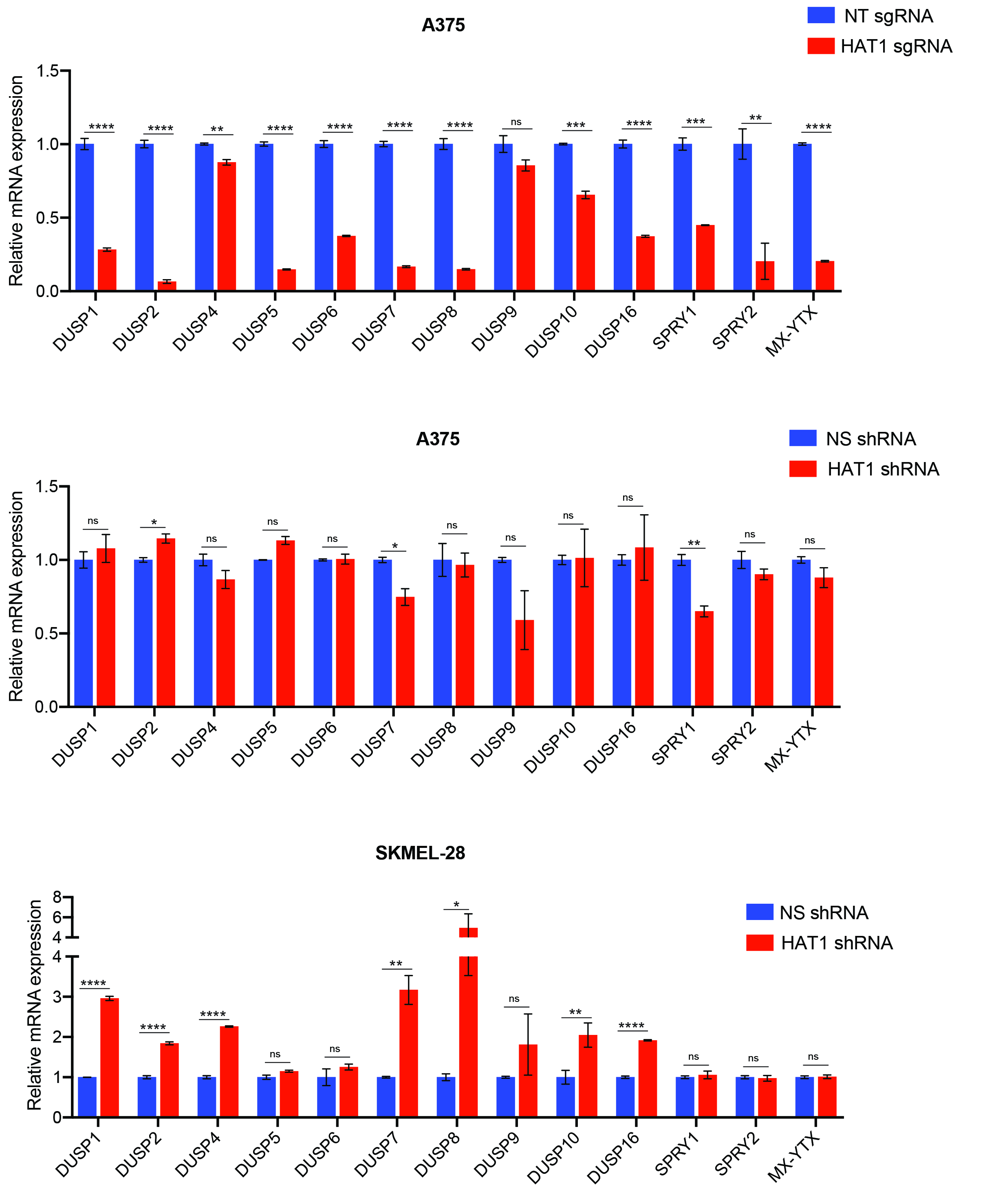

Supplement: Supplementary file 6 — Supplementary Figure 5 [file 41389_2020_228_MOESM6_ESM.tif]
