# Supplementary material for: Loss of HAT1 expression confers BRAFV600E inhibitor resistance to melanoma cells by activating MAPK signaling via IGF1R
Source: Oncogenesis. 2020 May 5;9(5):44. doi: 10.1038/s41389-020-0228-x (PMC7200761; doi:10.1038/s41389-020-0228-x)
Supplement: Supplementary file 7 — Supplementary Table 1 [file 41389_2020_228_MOESM7_ESM.docx]

**Supplementary Table 1.** Patient-derived melanoma sample information used for HAT1 expression analysis.

| **S.No.** | **Sample ID** | **Stage** | **Mutation** | **Treatment*** | **Response*** | **Samples analyzed** | **HAT1 protein expression in progressed samples** |
| --- | --- | --- | --- | --- | --- | --- | --- |
| Pair 1 | #1218 | IV M1c | V600E | Seq. BRAFi + MEKi | SD | Matched Pre- and progressed | ***Downregulated*** |
| Pair 2 | #1117 | IV M1c | V600E | Dual BRAFi + MEKi | PR | Matched Pre- and progressed | ***Downregulated***   |
| Pair 3 | #848 | IV M1c | V600E | Dual BRAFi + MEKi | PR | Matched Pre- and progressed | ***Downregulated*** |
| Pair 4 | #757 | IV M1c | V600E | BRAFi | PR   | Matched Pre- and progressed | ***Downregulated***  **** |
| Pair 5 | #0470 | IV M1c | V600K | BRAFi | PR | Matched Pre- and progressed | Upregulated |
| Pair 6 | #0959 | IV M1a | V600E | BRAFi | PR | Matched Pre- and progressed | ***Downregulated*** |
| Pair 7 | #0923 | IV M1c | V600E | Dual BRAFi + MEKi | PR   | Matched Pre- and progressed | Unchanged   |
| Pair 8 | #2132 | IV M1c | V600E | Dual BRAFi + MEKi | PR | Matched Pre- and progressed | Upregulated |
| Pair 9 | #1351 | IV M1a | V600E | Seq. BRAFi + MEKi | PR   | Matched Pre- and progressed | ***Downregulated *** |
| Pair 10 | #1577 | IV M1c | V600E | Dual BRAFi + MEKi | PR | Matched Pre- and progressed | Upregulated |
| Pair 11 | #1254 | IV M1c   | V600E   | BRAFi | PR | Matched Pre- and progressed | ***Downregulated *** |

*SD: Stable disease PR: Partial response
