# Supplementary material for: Loss of HAT1 expression confers BRAFV600E inhibitor resistance to melanoma cells by activating MAPK signaling via IGF1R
Source: Oncogenesis. 2020 May 5;9(5):44. doi: 10.1038/s41389-020-0228-x (PMC7200761; doi:10.1038/s41389-020-0228-x)
Supplement: Supplementary file 8 — Supplementary Table 2 [file 41389_2020_228_MOESM8_ESM.docx]

**Supplementary Table 2:** List of genes in indicated pathways along with fold change expression and P values. “1” represents for samples where fold change could not be measured.

| **RAS pathway Genes** |  |  |  |  |  |  |  |
| --- | --- | --- | --- | --- | --- | --- | --- |
| **Probe Name** | **Accession #** | **HAT1 sgRNA vs. NT** | **P value of: sample 3 vs. Reference** | **A375 HAT1 shRNA vs. NS** | **P value of: sample 2 vs. Reference** | **SKMEL-28 HAT1 shRNA vs. NS** | **P value of: sample 2 vs. Reference** |
| ABL1 | NM_005157.3 | 1 | 0.975 | 1.15 | 0.053 | -1.08 | 0.603 |
| AKT1 | NM_005163.2 | 1.63 | 4E-04 | 1.06 | 0.269 | -1.04 | 0.338 |
| AKT2 | NM_001626.2 | 1.09 | 0.196 | 1 | 0.93 | 1.6 | 0.001 |
| AKT3 | NM_181690.1 | 1.12 | 0.024 | 1.23 | 0.007 | 1.11 | 0.307 |
| ANGPT1 | NM_001146.3 | -1.01 | 0.94 | -1.79 | 0.014 | -1.57 | 8E-04 |
| BAD | NM_004322.3 | 1.36 | 0.002 | -1.03 | 0.627 | -1.27 | 0.089 |
| BCL2L1 | NM_138578.1 | -2.97 | 3E-05 | -1.08 | 0.334 | -1.01 | 0.942 |
| CALML3 | NM_005185.2 | 1 |  | 1 |  | 1 |  |
| CALML5 | NM_017422.4 | -1.01 | 0.423 | 1 |  | 1 |  |
| CALML6 | NM_138705.2 | 1 |  | 1 |  | 1.12 | 0.423 |
| CHUK | NM_001278.3 | -1.49 | 0.003 | 1.05 | 0.619 | -1.45 | 0.047 |
| CSF1R | NM_005211.2 | 1 |  | 1 |  | 1 |  |
| EFNA1 | NM_004428.2 | -1.03 | 0.835 | 1.28 | 0.122 | 1.63 | 0.423 |
| EFNA2 | NM_001405.3 | 1 |  | 1 |  | 1 |  |
| EFNA3 | NM_004952.4 | 1.5 | 0.02 | -1.02 | 0.89 | 4.36 | 0.002 |
| EFNA5 | NM_001962.2 | -1.34 | 0.103 | 1.23 | 0.389 | -1.37 | 0.149 |
| EGF | NM_001963.3 | 1.04 | 0.423 | 1.16 | 0.656 | 2.35 | 0.005 |
| EGFR | NM_201282.1 | -2.08 | 2E-04 | -1.24 | 0.126 | 1.43 | 0.299 |
| EPHA2 | NM_004431.2 | -4.43 | 6E-05 | -1.29 | 0.059 | -1.13 | 0.423 |
| ETS2 | NM_005239.4 | -1.63 | 0.038 | 1.18 | 0.169 | -1.1 | 0.217 |
| FASLG | NM_000639.1 | 1 |  | 1 |  | 1 |  |
| FGF1 | NM_033137.1 | 1 |  | 2.63 | 0.003 | -2.16 | 0.042 |
| FGF10 | NM_004465.1 | 1 |  | 1 |  | 1 |  |
| FGF11 | NM_004112.2 | 1.45 | 0.208 | 1.68 | 0.175 | 1.2 | 0.423 |
| FGF12 | NM_004113.4 | 2.94 | 8E-04 | 1.11 | 0.566 | 1 |  |
| FGF13 | NM_033642.1 | 2.9 | 0.003 | -1.03 | 0.683 | -1.35 | 0.013 |
| FGF14 | NM_004115.3 | -1.22 | 0.423 | 1.2 | 0.419 | 1.29 | 0.469 |
| FGF16 | NM_003868.1 | 1 |  | 1 |  | 1 |  |
| FGF17 | NM_003867.2 | -1.11 | 0.423 | 1 |  | 1 |  |
| FGF18 | NM_003862.1 | 1 |  | 1 |  | 1 |  |
| FGF19 | NM_005117.2 | -1.03 | 0.423 | 1 |  | 1 |  |
| FGF2 | NM_002006.4 | -1.92 | 0.005 | 1.1 | 0.162 | 1.34 | 0.012 |
| FGF20 | NM_019851.1 | 1 |  | 1 |  | 1 |  |
| FGF21 | NM_019113.2 | 1 |  | 1 |  | 1 |  |
| FGF22 | NM_020637.1 | -1.11 | 0.423 | 1 |  | -1.03 | 0.423 |
| FGF23 | NM_020638.2 | 1 |  | 1 |  | 1 |  |
| FGF3 | NM_005247.2 | 1 |  | 1 |  | 1 |  |
| FGF4 | NM_002007.2 | -1.03 | 0.423 | 1 |  | 1 |  |
| FGF5 | NM_004464.3 | 1 |  | 1 |  | 1 |  |
| FGF6 | NM_020996.1 | 1 |  | 1 |  | 1 |  |
| FGF7 | NM_002009.3 | -1.01 | 0.216 | 1 |  | 1 |  |
| FGF8 | NM_033163.3 | 1 |  | 1 |  | 1 |  |
| FGF9 | NM_002010.2 | -1.01 | 0.423 | 1 |  | 1 |  |
| FGFR1 | NM_015850.2 | -1.3 | 0.021 | -1.24 | 0.055 | -1.81 | 0.015 |
| FGFR2 | NM_000141.4 | 1 |  | 1 |  | 1 |  |
| FGFR3 | NM_022965.2 | 1.97 | 0.035 | -1.16 | 0.131 | 1.27 | 0.423 |
| FGFR4 | NM_002011.3 | 2.48 | 0.009 | 1.33 | 0.119 | -1.25 | 0.623 |
| FIGF | NM_004469.2 | 1 |  | 1 |  | 1.25 | 0.184 |
| FLT1 | NM_002019.4 | 1.11 | 0.689 | 1.07 | 0.025 | 1.18 | 0.394 |
| FOXO4 | NM_005938.2 | 1.23 | 0.265 | 1.38 | 0.026 | 1.56 | 0.013 |
| GNG12 | NM_018841.3 | 1.11 | 0.034 | 1.19 | 0.015 | -1.43 | 6E-04 |
| GNG4 | NM_004485.2 | 1.62 | 0.003 | 1.06 | 0.475 | 1.16 | 0.593 |
| GNG7 | NM_052847.1 | 1.9 | 0.04 | 1.04 | 0.423 | 2.04 | 0.189 |
| GNGT1 | NM_021955.3 | -1.09 | 0.745 | 1.17 | 0.423 | 1 |  |
| GRB2 | NM_002086.4 | -1.08 | 0.155 | 1.1 | 0.026 | -1.06 | 0.296 |
| GRIN1 | NM_000832.5 | 1 |  | 1 |  | 1 |  |
| GRIN2A | NM_000833.3 | 1 |  | 1 |  | 1 |  |
| GRIN2B | NM_000834.3 | 1 |  | 1 |  | 1 |  |
| HGF | NM_000601.4 | 1 |  | 1 |  | 1 |  |
| HRAS | NM_005343.2 | -1.46 | 0.052 | -1.07 | 0.396 | -1.38 | 0.157 |
| IGF1 | NM_000618.3 | -1.01 | 0.423 | -1.05 | 0.423 | 1 |  |
| IGF1R | NM_000875.2 | 2.24 | 0.004 | 1.09 | 0.054 | 1.67 | 2E-05 |
| IKBKB | NM_001556.1 | -1.32 | 0.241 | 1.12 | 0.57 | -1.32 | 0.375 |
| IKBKG | NM_003639.2 | -1.32 | 0.03 | -1.12 | 0.205 | -1.43 | 0.05 |
| KIT | NM_000222.1 | 1 |  | 1 |  | 1.11 | 0.423 |
| KITLG | NM_003994.4 | -1.04 | 0.854 | -1.22 | 0.144 | -1.99 | 0.02 |
| KRAS | NM_004985.3 | -1.27 | 0.059 | 1.16 | 0.15 | 1.08 | 0.43 |
| LAT | NM_001014987.1 | 1.14 | 0.52 | -1.04 | 0.743 | -1.01 | 0.423 |
| MAP2K1 | NM_002755.2 | -1.2 | 0.08 | -1.05 | 0.275 | 1.33 | 0.062 |
| MAP2K2 | NM_030662.2 | 1.58 | 0.002 | 1.16 | 0.081 | 1.24 | 0.022 |
| MAPK1 | NM_138957.2 | -1.03 | 0.519 | 1.14 | 0.033 | 1.16 | 0.005 |
| MAPK10 | NM_002753.2 | -1.01 | 0.423 | 1 |  | 1 |  |
| MAPK3 | NM_001040056.1 | 1.56 | 1E-04 | -1.25 | 0.003 | 1.28 | 0.035 |
| MAPK8 | NM_002750.2 | -1.74 | 0.003 | -1.01 | 0.893 | -1.07 | 0.534 |
| MAPK9 | NM_139068.2 | 1.36 | 9E-04 | 1.05 | 0.168 | 1.01 | 0.876 |
| MET | NM_000245.2 | -2.41 | 3E-06 | 1.01 | 0.859 | 3.11 | 4E-04 |
| MLLT4 | NM_005936.2 | -1.26 | 0.102 | 1.04 | 0.765 | -1.1 | 0.218 |
| NF1 | NM_000267.2 | 1.15 | 0.116 | 1.21 | 0.055 | 1.53 | 0.022 |
| NFKB1 | NM_003998.2 | 1.1 | 0.336 | -1.23 | 0.279 | 1.18 | 0.307 |
| NGF | NM_002506.2 | -1.16 | 0.423 | 1 |  | 1 |  |
| NGFR | NM_002507.1 | -69.44 | 2E-05 | 1.66 | 0.052 | 3.78 | 0.024 |
| NRAS | NM_002524.3 | -1.14 | 0.392 | -1.47 | 0.028 | -1.26 | 0.04 |
| PAK3 | NM_002578.2 | 1.15 | 0.494 | 1 |  | 1 |  |
| PAK7 | NM_177990.1 | -1.01 | 0.423 | 1 |  | 1 |  |
| PDGFA | NM_002607.5 | -2.78 | 0.027 | -1.66 | 0.158 | 1 |  |
| PDGFB | NM_033016.2 | 1 |  | 1 |  | 1 |  |
| PDGFC | NM_016205.1 | -1.06 | 0.728 | 1.05 | 0.816 | 1 |  |
| PDGFD | NM_025208.4 | 1 |  | 1.19 | 0.573 | -1.02 | 0.873 |
| PDGFRA | NM_006206.3 | 2.27 | 0.001 | 2.13 | 0.005 | 1 |  |
| PDGFRB | NM_002609.3 | 1.11 | 0.548 | 1.13 | 0.361 | 1 |  |
| PGF | NM_002632.5 | 1.39 | 0.214 | 1.17 | 0.632 | 1.64 | 0.183 |
| PIK3CA | NM_006218.2 | 1.09 | 0.2 | 1.13 | 0.303 | 1.47 | 0.003 |
| PIK3CB | NM_006219.1 | 1.46 | 0.017 | 1.12 | 0.288 | 1 | 0.937 |
| PIK3CD | NM_005026.3 | 1.32 | 5E-04 | -1.21 | 0.103 | 1.01 | 0.954 |
| PIK3CG | NM_002649.2 | 1 |  | 1 |  | 1 |  |
| PIK3R1 | NM_181504.2 | -1.29 | 0.133 | -1.09 | 0.459 | -1.23 | 0.207 |
| PIK3R2 | NM_005027.2 | 1.68 | 0.015 | 1.18 | 0.113 | 1.37 | 0.061 |
| PIK3R3 | NM_003629.3 | 2.2 | 0.027 | 1.29 | 0.088 | -1.22 | 3E-06 |
| PIK3R5 | NM_001142633.1 | -1.01 | 0.423 | 1 |  | 1 |  |
| PLA1A | NM_015900.2 | 1 |  | 1 |  | 1.07 | 0.093 |
| PLA2G10 | NM_003561.1 | 1 |  | 1 |  | 1 |  |
| PLA2G2A | NM_000300.2 | 1.04 | 0.423 | 1.1 | 0.713 | 1 |  |
| PLA2G3 | NM_015715.3 | -1.08 | 0.678 | -1.19 | 0.425 | 1.11 | 0.423 |
| PLA2G4A | NM_024420.2 | 1.21 | 0.456 | 1.33 | 0.029 | 1.73 | 0.013 |
| PLA2G4C | NM_003706.2 | 1 |  | 1 |  | 1.4 | 0.22 |
| PLA2G4E | NM_001206670.1 | 1 |  | 1 |  | 1 |  |
| PLA2G4F | NM_213600.2 | 1 |  | 1 |  | 1 |  |
| PLA2G5 | NM_000929.2 | 1 |  | 1 |  | 1 |  |
| PLAT | NM_000931.2 | -1.38 | 4E-04 | -1.13 | 0.076 | 1.41 | 5E-04 |
| PLCG2 | NM_002661.2 | -1.65 | 0.072 | 1.44 | 0.277 | -1.26 | 0.266 |
| PLD1 | NM_002662.3 | 3.65 | 0.001 | 1.34 | 0.048 | 2.33 | 0.002 |
| PRKACA | NM_002730.3 | 1.29 | 0.004 | 1.08 | 0.166 | 1.04 | 0.328 |
| PRKACB | NM_182948.2 | -1.37 | 0.012 | 1.01 | 0.885 | 1.27 | 0.116 |
| PRKACG | NM_002732.2 | 1 |  | 1 |  | 1 |  |
| PRKCA | NM_002737.2 | 1 | 0.992 | 1.13 | 0.165 | 1 |  |
| PRKCB | NM_212535.1 | 1 |  | 1 |  | 3.72 | 0.009 |
| PRKCG | NM_002739.3 | 1 |  | 1 |  | 1 |  |
| PRKX | NM_005044.1 | 1.44 | 0.372 | 1.48 | 0.139 | -1.24 | 0.131 |
| PTPN11 | NM_002834.3 | -1.35 | 0.006 | 1.05 | 0.046 | 1.18 | 0.063 |
| RAC1 | NM_198829.1 | -1.18 | 0.043 | -1.07 | 0.191 | 1 | 0.523 |
| RAC2 | NM_002872.3 | -1.31 | 0.286 | 1.04 | 0.423 | 1.18 | 0.467 |
| RAC3 | NM_005052.2 | 3.05 | 0.026 | -1.03 | 0.839 | 1.53 | 0.048 |
| RAF1 | NM_002880.2 | 1.13 | 0.171 | 1.17 | 0.048 | 1.17 | 0.033 |
| RASA4 | NM_001079877.2 | 2.36 | 0.009 | 1.01 | 0.97 | 2.19 | 0.054 |
| RASAL1 | NM_004658.1 | -1.19 | 0.47 | -1.2 | 0.545 | 1.34 | 0.216 |
| RASGRF1 | NM_153815.2 | -1.27 | 0.423 | 1 |  | 2.18 | 0.015 |
| RASGRF2 | NM_006909.1 | 1 |  | 1 |  | 1 |  |
| RASGRP1 | NM_005739.3 | 1 |  | 1 |  | 1 |  |
| RASGRP2 | NM_001098670.1 | 1 |  | 1 |  | 1 |  |
| RELA | NM_021975.3 | -1.08 | 0.342 | -1.12 | 0.185 | 1.59 | 0.021 |
| RHOA | NM_001664.2 | 1.25 | 0.01 | -1 | 0.91 | 1.21 | 0.086 |
| RIN1 | NM_004292.2 | 1.59 | 0.027 | 1.28 | 0.008 | 1.35 | 0.017 |
| RRAS2 | NM_001102669.2 | -1.68 | 0.057 | 1.34 | 0.023 | -1.03 | 0.622 |
| SHC1 | NM_183001.4 | 1.2 | 0.019 | 1.23 | 0.007 | 1.31 | 8E-05 |
| SHC2 | NM_012435.2 | 1.35 | 0.221 | 1 |  | 1 |  |
| SHC3 | NM_016848.5 | 1 |  | 1 |  | 1 |  |
| SHC4 | NM_203349.2 | 1.72 | 0.005 | 1.15 | 0.216 | 1.14 | 0.215 |
| SOS1 | NM_005633.2 | 1.27 | 0.047 | 1.08 | 0.385 | 1.15 | 0.105 |
| SOS2 | NM_006939.2 | -1.23 | 0.232 | -1.03 | 0.527 | -1.17 | 0.206 |
| TIAM1 | NM_003253.2 | 1.79 | 0.013 | -1.17 | 0.151 | 1.11 | 0.408 |
| VEGFA | NM_001025366.1 | -3.6 | 9E-05 | -1.02 | 0.744 | 1.87 | 0.013 |
| VEGFC | NM_005429.2 | 1.59 | 0.012 | -1.32 | 0.07 | 3.25 | 5E-04 |
|  |  |  |  |  |  |  |  |
| **MAPK Pathway Genes** | |  |  |  |  |  |  |
| **Probe Name** | **Accession #** | **HAT1 sgRNA vs. NT** | **P value of: sample 3 vs. Reference** | **A375 HAT1 shRNA vs. NS** | **P value of: sample 2 vs. Reference** | **SKMEL-28 HAT1 shRNA vs. NS** | **P value of: sample 2 vs. Reference** |
| AKT1 | NM_005163.2 | 1.63 | 4E-04 | 1.06 | 0.269 | -1.04 | 0.338 |
| AKT2 | NM_001626.2 | 1.09 | 0.196 | 1 | 0.93 | 1.6 | 0.001 |
| AKT3 | NM_181690.1 | 1.12 | 0.024 | 1.23 | 0.007 | 1.11 | 0.307 |
| BDNF | NM_170732.4 | -2.83 | 0.003 | 1.34 | 0.021 | 1 | 0.989 |
| BRAF | NM_004333.3 | -1.07 | 0.432 | 1.01 | 0.824 | 1.05 | 0.661 |
| CACNA1C | NM_199460.2 | 1 |  | 1 |  | 1 |  |
| CACNA1D | NM_000720.2 | -1.01 | 0.423 | 1.47 | 0.19 | -1.94 | 0.024 |
| CACNA1E | NM_000721.2 | 1 |  | 1 |  | 1 |  |
| CACNA1G | NM_198397.1 | -1.01 | 0.423 | 1 |  | 1.11 | 0.423 |
| CACNA1H | NM_021098.2 | 1 |  | 1 |  | 2.46 | 0.045 |
| CACNA2D1 | NM_000722.2 | 1.09 | 0.418 | 1.21 | 0.194 | 1 |  |
| CACNA2D2 | NM_001005505.1 | 1 |  | 1 |  | 1 |  |
| CACNA2D3 | NM_018398.2 | 2.24 | 0.067 | -1.5 | 0.278 | 1 |  |
| CACNA2D4 | NM_001005737.1 | 1 |  | 1 |  | 1 |  |
| CACNB2 | NM_000724.3 | 1 |  | 1 |  | 1 |  |
| CACNB3 | NM_000725.2 | 1.57 | 0.179 | -1.04 | 0.668 | 1.52 | 0.199 |
| CACNB4 | NM_001005747.2 | 1 |  | -1.05 | 0.423 | 1.05 | 0.817 |
| CACNG1 | NM_000727.2 | 1 |  | 1 |  | 1 |  |
| CACNG4 | NM_014405.2 | 1 |  | 1 |  | 1 |  |
| CACNG6 | NM_145814.1 | 1.04 | 0.882 | 1.21 | 0.539 | 1 |  |
| CASP3 | NM_032991.2 | -1.33 | 0.008 | 1.12 | 0.015 | -1.28 | 0.012 |
| CD14 | NM_000591.2 | 1 |  | -1.12 | 0.423 | 1 |  |
| CDC25B | NM_021873.2 | 1.98 | 8E-05 | 1.03 | 0.585 | -1.27 | 0.037 |
| CHUK | NM_001278.3 | -1.49 | 0.003 | 1.05 | 0.619 | -1.45 | 0.047 |
| DAXX | NM_001350.3 | 1.24 | 0.107 | -1.02 | 0.611 | -1.17 | 0.11 |
| DDIT3 | NM_004083.4 | -7.47 | 8E-05 | 1.35 | 0.014 | 1.8 | 0.003 |
| DUSP10 | NM_144728.2 | -1.19 | 0.084 | 1.43 | 0.008 | 1.52 | 0.03 |
| DUSP2 | NM_004418.3 | -1.52 | 0.029 | 1.01 | 0.878 | 1 |  |
| DUSP4 | NM_057158.2 | 2.04 | 2E-04 | 1.04 | 0.166 | 1.18 | 3E-04 |
| DUSP5 | NM_004419.3 | -4.42 | 0.002 | 1.06 | 0.378 | 1.17 | 0.063 |
| DUSP6 | NM_001946.2 | -1.66 | 3E-07 | 1 | 0.967 | -2.35 | 6E-04 |
| DUSP8 | NM_004420.2 | -1.09 | 0.786 | -1.01 | 0.951 | 1.11 | 0.423 |
| EGF | NM_001963.3 | 1.04 | 0.423 | 1.16 | 0.656 | 2.35 | 0.005 |
| EGFR | NM_201282.1 | -2.08 | 2E-04 | -1.24 | 0.126 | 1.43 | 0.299 |
| FAS | NM_152876.1 | 1.14 | 0.29 | 1.21 | 0.101 | -1.04 | 0.927 |
| FASLG | NM_000639.1 | 1 |  | 1 |  | 1 |  |
| FGF1 | NM_033137.1 | 1 |  | 2.63 | 0.003 | -2.16 | 0.042 |
| FGF10 | NM_004465.1 | 1 |  | 1 |  | 1 |  |
| FGF11 | NM_004112.2 | 1.45 | 0.208 | 1.68 | 0.175 | 1.2 | 0.423 |
| FGF12 | NM_004113.4 | 2.94 | 8E-04 | 1.11 | 0.566 | 1 |  |
| FGF13 | NM_033642.1 | 2.9 | 0.003 | -1.03 | 0.683 | -1.35 | 0.013 |
| FGF14 | NM_004115.3 | -1.22 | 0.423 | 1.2 | 0.419 | 1.29 | 0.469 |
| FGF16 | NM_003868.1 | 1 |  | 1 |  | 1 |  |
| FGF17 | NM_003867.2 | -1.11 | 0.423 | 1 |  | 1 |  |
| FGF18 | NM_003862.1 | 1 |  | 1 |  | 1 |  |
| FGF19 | NM_005117.2 | -1.03 | 0.423 | 1 |  | 1 |  |
| FGF2 | NM_002006.4 | -1.92 | 0.005 | 1.1 | 0.162 | 1.34 | 0.012 |
| FGF20 | NM_019851.1 | 1 |  | 1 |  | 1 |  |
| FGF21 | NM_019113.2 | 1 |  | 1 |  | 1 |  |
| FGF22 | NM_020637.1 | -1.11 | 0.423 | 1 |  | -1.03 | 0.423 |
| FGF23 | NM_020638.2 | 1 |  | 1 |  | 1 |  |
| FGF3 | NM_005247.2 | 1 |  | 1 |  | 1 |  |
| FGF4 | NM_002007.2 | -1.03 | 0.423 | 1 |  | 1 |  |
| FGF5 | NM_004464.3 | 1 |  | 1 |  | 1 |  |
| FGF6 | NM_020996.1 | 1 |  | 1 |  | 1 |  |
| FGF7 | NM_002009.3 | -1.01 | 0.216 | 1 |  | 1 |  |
| FGF8 | NM_033163.3 | 1 |  | 1 |  | 1 |  |
| FGF9 | NM_002010.2 | -1.01 | 0.423 | 1 |  | 1 |  |
| FGFR1 | NM_015850.2 | -1.3 | 0.021 | -1.24 | 0.055 | -1.81 | 0.015 |
| FGFR2 | NM_000141.4 | 1 |  | 1 |  | 1 |  |
| FGFR3 | NM_022965.2 | 1.97 | 0.035 | -1.16 | 0.131 | 1.27 | 0.423 |
| FGFR4 | NM_002011.3 | 2.48 | 0.009 | 1.33 | 0.119 | -1.25 | 0.623 |
| FLNA | NM_001456.3 | -1.21 | 7E-04 | -1.05 | 0.075 | 1.16 | 0.006 |
| FLNC | NM_001127487.1 | 1.18 | 0.423 | 1 |  | 1 |  |
| FOS | NM_005252.2 | -1.27 | 0.026 | 1.05 | 0.68 | -2.02 | 0.011 |
| GADD45A | NM_001924.2 | -5.98 | 7E-04 | 1.2 | 0.095 | 1.52 | 0.004 |
| GADD45B | NM_015675.2 | -6.48 | 2E-04 | -1.65 | 0.11 | 2.17 | 0.262 |
| GADD45G | NM_006705.3 | 1 |  | 1 |  | 1 |  |
| GNG12 | NM_018841.3 | 1.11 | 0.034 | 1.19 | 0.015 | -1.43 | 6E-04 |
| GRB2 | NM_002086.4 | -1.08 | 0.155 | 1.1 | 0.026 | -1.06 | 0.296 |
| HSPA1A | NM_005345.5 | 1.15 | 0.523 | -1.11 | 0.345 | -1.27 | 0.014 |
| HSPA2 | NM_021979.3 | 1.77 | 0.099 | -1.09 | 0.677 | 1.68 | 0.005 |
| HSPA6 | NM_002155.3 | 1.14 | 0.282 | 1.12 | 0.508 | -1.3 | 0.285 |
| HSPB1 | NM_001540.3 | 1.94 | 0.029 | -1 | 0.98 | 2.19 | 0.005 |
| HRAS | NM_005343.2 | -1.46 | 0.052 | -1.07 | 0.396 | -1.38 | 0.157 |
| IKBKB | NM_001556.1 | -1.32 | 0.241 | 1.12 | 0.57 | -1.32 | 0.375 |
| IKBKG | NM_003639.2 | -1.32 | 0.03 | -1.12 | 0.205 | -1.43 | 0.05 |
| IL1A | NM_000575.3 | 1.62 | 8E-04 | -1.47 | 0.025 | 1 |  |
| IL1B | NM_000576.2 | 2.03 | 4E-04 | -1.54 | 0.008 | -3.07 | 5E-04 |
| IL1R1 | NM_000877.2 | 1.02 | 0.869 | 1 |  | 1.27 | 0.423 |
| IL1R2 | NM_173343.1 | 1.06 | 0.423 | 1 |  | 1 |  |
| JUN | NM_002228.3 | -14.1 | 0.001 | 1.12 | 0.709 | 1.44 | 0.212 |
| KRAS | NM_004985.3 | -1.27 | 0.059 | 1.16 | 0.15 | 1.08 | 0.43 |
| MAP2K1 | NM_002755.2 | -1.2 | 0.08 | -1.05 | 0.275 | 1.33 | 0.062 |
| MAP2K2 | NM_030662.2 | 1.58 | 0.002 | 1.16 | 0.081 | 1.24 | 0.022 |
| MAP2K4 | NM_003010.2 | -1.16 | 0.163 | 1.1 | 0.381 | 1.27 | 0.053 |
| MAP2K6 | NM_002758.3 | 6.25 | 0.002 | 1.09 | 0.196 | 1.88 | 0.025 |
| MAP3K1 | NM_005921.1 | -1.08 | 0.336 | -1.02 | 0.711 | -1.1 | 0.248 |
| MAP3K12 | NM_006301.2 | 1.32 | 0.032 | 1.13 | 0.5 | 1.21 | 0.51 |
| MAP3K13 | NM_004721.3 | 1.3 | 0.379 | 1.53 | 0.211 | 1.74 | 0.174 |
| MAP3K14 | NM_003954.1 | -1.54 | 0.027 | -1.19 | 0.291 | 1.15 | 0.703 |
| MAP3K5 | NM_005923.3 | 1.91 | 0.089 | -1.45 | 0.122 | -1.24 | 0.557 |
| MAP3K8 | NM_005204.2 | -1.01 | 0.423 | 1.11 | 0.423 | 2.5 | 0.021 |
| MAPK1 | NM_138957.2 | -1.03 | 0.519 | 1.14 | 0.033 | 1.16 | 0.005 |
| MAPK10 | NM_002753.2 | -1.01 | 0.423 | 1 |  | 1 |  |
| MAPK12 | NM_002969.3 | 1.12 | 0.56 | 1.1 | 0.711 | 1.21 | 0.362 |
| MAPK3 | NM_001040056.1 | 1.56 | 1E-04 | -1.25 | 0.003 | 1.28 | 0.035 |
| MAPK8 | NM_002750.2 | -1.74 | 0.003 | -1.01 | 0.893 | -1.07 | 0.534 |
| MAPK8IP1 | NM_005456.2 | 1.31 | 0.213 | 1.11 | 0.554 | 1 |  |
| MAPK8IP2 | NM_012324.2 | 1 |  | 1 |  | -1.14 | 0.626 |
| MAPK9 | NM_139068.2 | 1.36 | 9E-04 | 1.05 | 0.168 | 1.01 | 0.876 |
| MAPT | NM_016834.3 | 1.55 | 0.218 | 1.56 | 0.281 | 2.76 | 0.024 |
| MECOM | NM_005241.2 | -1.6 | 0.239 | 1.14 | 0.624 | 1 |  |
| MYC | NM_002467.3 | -3.07 | 0.015 | 1.03 | 0.748 | 1.4 | 0.029 |
| NF1 | NM_000267.2 | 1.15 | 0.116 | 1.21 | 0.055 | 1.53 | 0.022 |
| NFATC1 | NM_172389.1 | -1.07 | 0.822 | 2.2 | 0.085 | -1.07 | 0.184 |
| NFKB1 | NM_003998.2 | 1.1 | 0.336 | -1.23 | 0.279 | 1.18 | 0.307 |
| NGF | NM_002506.2 | -1.16 | 0.423 | 1 |  | 1 |  |
| NR4A1 | NM_173157.1 | -1.33 | 0.047 | -1.16 | 0.072 | 1.76 | 0.003 |
| NRAS | NM_002524.3 | -1.14 | 0.392 | -1.47 | 0.028 | -1.26 | 0.04 |
| NTF3 | NM_002527.4 | 1 |  | 1 |  | 1 |  |
| NTRK1 | NM_001012331.1 | -3.34 | 0.042 | 1 |  | 1 |  |
| NTRK2 | NM_001007097.1 | 1 |  | -1.04 | 0.423 | 1.38 | 0.23 |
| PDGFA | NM_002607.5 | -2.78 | 0.027 | -1.66 | 0.158 | 1 |  |
| PDGFB | NM_033016.2 | 1 |  | 1 |  | 1 |  |
| PDGFRA | NM_006206.3 | 2.27 | 0.001 | 2.13 | 0.005 | 1 |  |
| PDGFRB | NM_002609.3 | 1.11 | 0.548 | 1.13 | 0.361 | 1 |  |
| PLA2G4A | NM_024420.2 | 1.21 | 0.456 | 1.33 | 0.029 | 1.73 | 0.013 |
| PLA2G4C | NM_003706.2 | 1 |  | 1 |  | 1.4 | 0.22 |
| PLA2G4E | NM_001206670.1 | 1 |  | 1 |  | 1 |  |
| PLA2G4F | NM_213600.2 | 1 |  | 1 |  | 1 |  |
| PPP3CA | NM_000944.4 | -1.14 | 0.24 | 1.07 | 0.194 | 1.15 | 0.277 |
| PPP3CB | NM_001142354.1 | 1.78 | 0.072 | 1.07 | 0.68 | 1.19 | 0.336 |
| PPP3CC | NM_005605.3 | -1.13 | 0.491 | -1.35 | 0.081 | 1.01 | 0.939 |
| PPP3R1 | NM_000945.3 | -1.35 | 0.014 | 1.15 | 0.061 | 1.08 | 0.1 |
| PPP3R2 | NM_147180.2 | 1 |  | 1 |  | 1 |  |
| PRKACA | NM_002730.3 | 1.29 | 0.004 | 1.08 | 0.166 | 1.04 | 0.328 |
| PRKACB | NM_182948.2 | -1.37 | 0.012 | 1.01 | 0.885 | 1.27 | 0.116 |
| PRKACG | NM_002732.2 | 1 |  | 1 |  | 1 |  |
| PRKCA | NM_002737.2 | 1 | 0.992 | 1.13 | 0.165 | 1 |  |
| PRKCB | NM_212535.1 | 1 |  | 1 |  | 3.72 | 0.009 |
| PRKCG | NM_002739.3 | 1 |  | 1 |  | 1 |  |
| PRKX | NM_005044.1 | 1.44 | 0.372 | 1.48 | 0.139 | -1.24 | 0.131 |
| PTPN5 | NM_001039970.1 | 1 |  | 1 |  | 1 |  |
| PTPRR | NM_001207015.1 | -5.63 | 0.007 | 1 |  | 3.35 | 3E-04 |
| RAC1 | NM_198829.1 | -1.18 | 0.043 | -1.07 | 0.191 | 1 | 0.523 |
| RAC2 | NM_002872.3 | -1.31 | 0.286 | 1.04 | 0.423 | 1.18 | 0.467 |
| RAC3 | NM_005052.2 | 3.05 | 0.026 | -1.03 | 0.839 | 1.53 | 0.048 |
| RAF1 | NM_002880.2 | 1.13 | 0.171 | 1.17 | 0.048 | 1.17 | 0.033 |
| RASGRF1 | NM_153815.2 | -1.27 | 0.423 | 1 |  | 2.18 | 0.015 |
| RASGRF2 | NM_006909.1 | 1 |  | 1 |  | 1 |  |
| RASGRP1 | NM_005739.3 | 1 |  | 1 |  | 1 |  |
| RASGRP2 | NM_001098670.1 | 1 |  | 1 |  | 1 |  |
| RELA | NM_021975.3 | -1.08 | 0.342 | -1.12 | 0.185 | 1.59 | 0.021 |
| RPS6KA5 | NM_004755.2 | 1.09 | 0.043 | 1.16 | 0.276 | -1.09 | 0.157 |
| RPS6KA6 | NM_014496.1 | -1.03 | 0.423 | 1 |  | 1 |  |
| RRAS2 | NM_001102669.2 | -1.68 | 0.057 | 1.34 | 0.023 | -1.03 | 0.622 |
| SOS1 | NM_005633.2 | 1.27 | 0.047 | 1.08 | 0.385 | 1.15 | 0.105 |
| SOS2 | NM_006939.2 | -1.23 | 0.232 | -1.03 | 0.527 | -1.17 | 0.206 |
| STMN1 | NM_203401.1 | 2.08 | 8E-04 | -1.04 | 0.101 | -1.04 | 0.161 |
| TGFB1 | NM_000660.3 | 1.13 | 0.05 | 1.26 | 0.022 | 14.55 | 0.003 |
| TGFB2 | NM_003238.2 | 1.18 | 0.214 | -1.13 | 0.251 | -1.01 | 0.423 |
| TGFB3 | NM_003239.2 | 1.41 | 0.173 | 1.48 | 0.121 | 1.65 | 0.201 |
| TGFBR2 | NM_001024847.1 | 1.32 | 0.04 | -1.83 | 0.014 | -1.23 | 0.229 |
| TNF | NM_000594.2 | -1.16 | 0.423 | 1 |  | 1 |  |
| TP53 | NM_000546.2 | 2.09 | 3E-06 | -1.12 | 0.149 | -1.08 | 0.286 |
| ZAK | NM_016653.2 | -1.07 | 0.681 | -1.01 | 0.941 | 1.35 | 0.02 |
|  |  |  |  |  |  |  |  |
| **TGF-β Pathway Genes** | |  |  |  |  |  |  |
| **Probe Name** | **Accession #** | **HAT1 sgRNA vs. NT** | **P value of: sample 3 vs. Reference** | **A375 HAT1 shRNA vs. NS** | **P value of: sample 2 vs. Reference** | **SKMEL-28 HAT1 shRNA vs. NS** | **P value of: sample 2 vs. Reference** |
| ACVR1B | NM_004302.3 | -1.02 | 0.94 | -1.04 | 0.735 | 1.03 | 0.897 |
| ACVR1C | NM_145259.2 | 1 |  | 1 |  | 1.59 | 0.029 |
| ACVR2A | NM_001616.3 | -1.52 | 0.009 | -1.07 | 0.724 | 1.16 | 0.48 |
| AMH | NM_000479.3 | 1 |  | -1.21 | 0.294 | 1 |  |
| BAMBI | NM_012342.2 | 3.11 | 0.005 | -1.15 | 0.004 | 1.38 | 3E-04 |
| BMP2 | NM_001200.2 | -2.03 | 0.007 | -1.17 | 0.132 | -1.13 | 0.387 |
| BMP4 | NM_001202.2 | 1.43 | 0.07 | -1.9 | 0.088 | 1 |  |
| BMP5 | NM_021073.2 | 1 |  | 1 |  | 1 |  |
| BMP6 | NM_001718.2 | -1.05 | 0.775 | 1.3 | 0.132 | 1 |  |
| BMP7 | NM_001719.1 | 1.02 | 0.571 | 1.02 | 0.949 | 1.73 | 0.002 |
| BMP8A | NM_181809.3 | 1.02 | 0.571 | 1 |  | 1 |  |
| BMPR1B | NM_001203.1 | -1.93 | 0.226 | -1.11 | 0.589 | 1.63 | 0.005 |
| CDKN2B | NM_004936.3 | 1 |  | 1 |  | -1.09 | 0.484 |
| CREBBP | NM_004380.2 | 1.17 | 0.364 | -1.46 | 0.238 | -1.22 | 0.321 |
| CUL1 | NM_003592.2 | -1.47 | 0.004 | -1.02 | 0.733 | -1 | 0.935 |
| E2F5 | NM_001951.3 | -1.43 | 0.007 | 1.31 | 0.041 | 2.48 | 0.006 |
| EP300 | NM_001429.2 | -1.06 | 0.439 | 1.1 | 0.153 | -1.03 | 0.558 |
| FST | NM_006350.2 | -5.15 | 6E-05 | 1.44 | 0.006 | 1 |  |
| GDF6 | NM_001001557.2 | 1 |  | 1 |  | 1 |  |
| ID1 | NM_002165.2 | 1.91 | 0.004 | -1.18 | 0.034 | -1.5 | 0.075 |
| ID2 | NM_002166.4 | 1.49 | 0.404 | -2.06 | 0.017 | -1.01 | 0.954 |
| ID4 | NM_001546.2 | 1 |  | 1.49 | 0.306 | -1.67 | 0.305 |
| IFNG | NM_000619.2 | 1 |  | 1 |  | 1 |  |
| INHBA | NM_002192.2 | -1.26 | 0.001 | -1.45 | 4E-04 | -1.03 | 0.423 |
| INHBB | NM_002193.2 | 1 |  | 1 |  | 1 |  |
| LEFTY1 | NM_020997.2 | 1 |  | 1.04 | 0.423 | 1.11 | 0.423 |
| LEFTY2 | NM_003240.2 | 1 |  | 1 |  | 1 |  |
| LTBP1 | NM_000627.3 | 1.74 | 0.004 | 1.38 | 0.157 | 2.73 | 0.005 |
| MAPK1 | NM_138957.2 | -1.03 | 0.519 | 1.14 | 0.033 | 1.16 | 0.005 |
| MAPK3 | NM_001040056.1 | 1.56 | 1E-04 | -1.25 | 0.003 | 1.28 | 0.035 |
| MYC | NM_002467.3 | -3.07 | 0.015 | 1.03 | 0.748 | 1.4 | 0.029 |
| NODAL | NM_018055.3 | 1 |  | 1 |  | 1 |  |
| NOG | NM_005450.4 | -5.57 | 3E-05 | 1 |  | 1.24 | 0.423 |
| PITX2 | NM_000325.5 | 1.1 | 0.442 | -1.03 | 0.423 | 1 |  |
| PPP2CB | NM_001009552.1 | -1.11 | 0.143 | -1.08 | 0.113 | 1.15 | 0.16 |
| PPP2R1A | NM_014225.3 | 1.65 | 7E-04 | 1.11 | 0.097 | 1.48 | 0.011 |
| RBX1 | NM_014248.2 | 1.18 | 0.016 | -1.09 | 0.055 | 1.07 | 0.031 |
| RHOA | NM_001664.2 | 1.25 | 0.01 | -1 | 0.91 | 1.21 | 0.086 |
| SKP1 | NM_170679.2 | 1.26 | 0.012 | 1.07 | 0.13 | 1.51 | 3E-04 |
| SKP2 | NM_005983.2 | 1.47 | 0.003 | -1.06 | 0.148 | -1.61 | 0.011 |
| SMAD2 | NM_001003652.1 | 1.06 | 0.325 | 1.07 | 0.409 | 1.11 | 0.267 |
| SMAD3 | NM_005902.3 | 2.35 | 5E-05 | -1.09 | 0.234 | 1.64 | 9E-04 |
| SMAD4 | NM_005359.3 | 1.18 | 0.178 | 1.05 | 0.522 | 1.22 | 0.023 |
| SP1 | NM_003109.1 | 1.1 | 0.332 | -1.01 | 0.935 | 1.16 | 0.485 |
| TFDP1 | NM_007111.4 | 1.05 | 0.134 | -1.26 | 0.013 | -2.27 | 0.003 |
| TGFB1 | NM_000660.3 | 1.13 | 0.05 | 1.26 | 0.022 | 14.55 | 0.003 |
| TGFB2 | NM_003238.2 | 1.18 | 0.214 | -1.13 | 0.251 | -1.01 | 0.423 |
| TGFB3 | NM_003239.2 | 1.41 | 0.173 | 1.48 | 0.121 | 1.65 | 0.201 |
| TGFBR2 | NM_001024847.1 | 1.32 | 0.04 | -1.83 | 0.014 | -1.23 | 0.229 |
| THBS1 | NM_003246.2 | -1.52 | 0.002 | -1.39 | 0.002 | 1.25 | 0.479 |
| TNF | NM_000594.2 | -1.16 | 0.423 | 1 |  | 1 |  |
|  |  |  |  |  |  |  |  |
| **Wnt Pathway Genes** | |  |  |  |  |  |  |
| **Probe Name** | **Accession #** | **HAT1 sgRNA vs. NT** | **P value of: sample 3 vs. Reference** | **A375 HAT1 shRNA vs. NS** | **P value of: sample 2 vs. Reference** | **SKMEL-28 HAT1 shRNA vs. NS** | **P value of: sample 2 vs. Reference** |
| APC | NM_000038.3 | 1.25 | 0.096 | 1.13 | 0.17 | 1.14 | 0.315 |
| AXIN1 | NM_181050.1 | -1.04 | 0.825 | -1.06 | 0.646 | -2.2 | 3E-04 |
| AXIN2 | NM_004655.3 | -1.01 | 0.423 | -1.32 | 0.369 | -1.25 | 0.123 |
| BAMBI | NM_012342.2 | 3.11 | 0.005 | -1.15 | 0.004 | 1.38 | 3E-04 |
| CAMK2B | NM_001220.3 | 1 |  | 1 |  | 1.07 | 0.571 |
| CCND1 | NM_053056.2 | -1.31 | 0.001 | -1.18 | 0.007 | -1.53 | 6E-04 |
| CCND2 | NM_001759.2 | -1.45 | 0.168 | 1 |  | 1 |  |
| CCND3 | NM_001760.2 | 1.01 | 0.942 | -1.03 | 0.729 | -1.08 | 0.599 |
| CREBBP | NM_004380.2 | 1.17 | 0.364 | -1.46 | 0.238 | -1.22 | 0.321 |
| CTNNB1 | NM_001904.3 | 1.3 | 2E-04 | 1.07 | 0.079 | 1.12 | 0.035 |
| CUL1 | NM_003592.2 | -1.47 | 0.004 | -1.02 | 0.733 | -1 | 0.935 |
| CXXC4 | NM_025212.1 | 1 |  | 1 |  | 1 |  |
| DKK1 | NM_012242.2 | -2.38 | 0.007 | -1.6 | 0.01 | -2.19 | 0.101 |
| DKK2 | NM_014421.2 | -1.45 | 0.298 | -1.31 | 0.111 | -1.18 | 0.423 |
| DKK4 | NM_014420.2 | 1 |  | 1 |  | 1 |  |
| EP300 | NM_001429.2 | -1.06 | 0.439 | 1.1 | 0.153 | -1.03 | 0.558 |
| FOSL1 | NM_005438.2 | -2.46 | 9E-07 | -1.23 | 0.013 | -1.76 | 0.008 |
| FZD10 | NM_007197.2 | 1 |  | 1 |  | 1 |  |
| FZD2 | NM_001466.2 | 1 | 0.999 | 1.74 | 0.072 | 1.57 | 0.06 |
| FZD3 | NM_017412.2 | 1.08 | 0.692 | 1.35 | 0.027 | 1.03 | 0.872 |
| FZD7 | NM_003507.1 | 1.29 | 0.231 | 1.09 | 0.44 | 1.52 | 0.03 |
| FZD8 | NM_031866.1 | 1.48 | 0.102 | -1.19 | 0.222 | 1 |  |
| FZD9 | NM_003508.2 | 2 | 0.008 | 1.04 | 0.903 | 1 |  |
| GPC4 | NM_001448.2 | 1 |  | 1.79 | 0.016 | 1.88 | 0.137 |
| GSK3B | NM_002093.2 | 1.72 | 3E-04 | 1.12 | 0.034 | 1.43 | 0.023 |
| JUN | NM_002228.3 | -14.1 | 0.001 | 1.12 | 0.709 | 1.44 | 0.212 |
| LEF1 | NM_016269.3 | -1.26 | 0.066 | -1.36 | 0.025 | 1.05 | 0.368 |
| MAPK10 | NM_002753.2 | -1.01 | 0.423 | 1 |  | 1 |  |
| MAPK8 | NM_002750.2 | -1.74 | 0.003 | -1.01 | 0.893 | -1.07 | 0.534 |
| MAPK9 | NM_139068.2 | 1.36 | 9E-04 | 1.05 | 0.168 | 1.01 | 0.876 |
| MMP7 | NM_002423.3 | 1 |  | 1 |  | 1 |  |
| MYC | NM_002467.3 | -3.07 | 0.015 | 1.03 | 0.748 | 1.4 | 0.029 |
| NFATC1 | NM_172389.1 | -1.07 | 0.822 | 2.2 | 0.085 | -1.07 | 0.184 |
| NKD1 | NM_033119.3 | 1 |  | 1 |  | 1 |  |
| PLCB1 | NM_182734.1 | 1.01 | 0.92 | 1.24 | 0.335 | 1.31 | 0.025 |
| PLCB4 | NM_000933.3 | 1.83 | 0.007 | -1.28 | 0.121 | -1.17 | 0.054 |
| PPP3CA | NM_000944.4 | -1.14 | 0.24 | 1.07 | 0.194 | 1.15 | 0.277 |
| PPP3CB | NM_001142354.1 | 1.78 | 0.072 | 1.07 | 0.68 | 1.19 | 0.336 |
| PPP3CC | NM_005605.3 | -1.13 | 0.491 | -1.35 | 0.081 | 1.01 | 0.939 |
| PPP3R1 | NM_000945.3 | -1.35 | 0.014 | 1.15 | 0.061 | 1.08 | 0.1 |
| PPP3R2 | NM_147180.2 | 1 |  | 1 |  | 1 |  |
| PRKACA | NM_002730.3 | 1.29 | 0.004 | 1.08 | 0.166 | 1.04 | 0.328 |
| PRKACB | NM_182948.2 | -1.37 | 0.012 | 1.01 | 0.885 | 1.27 | 0.116 |
| PRKACG | NM_002732.2 | 1 |  | 1 |  | 1 |  |
| PRKCA | NM_002737.2 | 1 | 0.992 | 1.13 | 0.165 | 1 |  |
| PRKCB | NM_212535.1 | 1 |  | 1 |  | 3.72 | 0.009 |
| PRKCG | NM_002739.3 | 1 |  | 1 |  | 1 |  |
| PRKX | NM_005044.1 | 1.44 | 0.372 | 1.48 | 0.139 | -1.24 | 0.131 |
| RAC1 | NM_198829.1 | -1.18 | 0.043 | -1.07 | 0.191 | 1 | 0.523 |
| RAC2 | NM_002872.3 | -1.31 | 0.286 | 1.04 | 0.423 | 1.18 | 0.467 |
| RAC3 | NM_005052.2 | 3.05 | 0.026 | -1.03 | 0.839 | 1.53 | 0.048 |
| RBX1 | NM_014248.2 | 1.18 | 0.016 | -1.09 | 0.055 | 1.07 | 0.031 |
| RHOA | NM_001664.2 | 1.25 | 0.01 | -1 | 0.91 | 1.21 | 0.086 |
| SFRP1 | NM_003012.3 | 2.03 | 9E-05 | 1.03 | 0.056 | -1.18 | 0.036 |
| SFRP2 | NM_003013.2 | 1 |  | 1 |  | 1 |  |
| SFRP4 | NM_003014.2 | 1 |  | 1 |  | 1 |  |
| SKP1 | NM_170679.2 | 1.26 | 0.012 | 1.07 | 0.13 | 1.51 | 3E-04 |
| SMAD3 | NM_005902.3 | 2.35 | 5E-05 | -1.09 | 0.234 | 1.64 | 9E-04 |
| SMAD4 | NM_005359.3 | 1.18 | 0.178 | 1.05 | 0.522 | 1.22 | 0.023 |
| SOST | NM_025237.2 | 1 |  | 1 |  | 1 |  |
| SOX17 | NM_022454.3 | -1.14 | 0.5 | 1 |  | 1 |  |
| TBL1XR1 | NM_024665.4 | -1.07 | 0.22 | 1.14 | 0.002 | 1.2 | 0.015 |
| TCF7L1 | NM_031283.1 | 4.21 | 0.002 | -1.29 | 0.288 | 1.42 | 0.053 |
| TP53 | NM_000546.2 | 2.09 | 3E-06 | -1.12 | 0.149 | -1.08 | 0.286 |
| WIF1 | NM_007191.2 | 1 |  | 1 |  | 1 |  |
| WNT10A | NM_025216.2 | -1.01 | 0.423 | 1 |  | 1 |  |
| WNT10B | NM_003394.2 | -1.95 | 0.015 | 1.32 | 0.105 | 2.66 | 0.035 |
| WNT11 | NM_004626.2 | 1 |  | 1 |  | 1 |  |
| WNT16 | NM_057168.1 | 1.18 | 0.423 | 1 |  | 1 |  |
| WNT2 | NM_003391.2 | 1 |  | 1 |  | 1 |  |
| WNT2B | NM_024494.1 | 1.09 | 0.243 | 1 |  | -1.06 | 0.423 |
| WNT3 | NM_030753.3 | -1.18 | 0.291 | 1.35 | 0.107 | 1.24 | 0.423 |
| WNT4 | NM_030761.3 | 1 |  | 1 |  | 1 |  |
| WNT5A | NM_003392.3 | 2.02 | 2E-05 | -2.06 | 6E-04 | 2.52 | 0.052 |
| WNT5B | NM_032642.2 | 1 |  | 1 |  | 1 |  |
| WNT6 | NM_006522.3 | 1.07 | 0.723 | 1 |  | 1.11 | 0.423 |
| WNT7A | NM_004625.3 | -1.02 | 0.854 | -1.04 | 0.715 | -1.03 | 0.831 |
| WNT7B | NM_058238.1 | -1.1 | 0.648 | 1 |  | 1 |  |
