# Supplementary material for: Loss of HAT1 expression confers BRAFV600E inhibitor resistance to melanoma cells by activating MAPK signaling via IGF1R
Source: Oncogenesis. 2020 May 5;9(5):44. doi: 10.1038/s41389-020-0228-x (PMC7200761; doi:10.1038/s41389-020-0228-x)
Supplement: Supplementary file 9 — Supplementary Table 3 [file 41389_2020_228_MOESM9_ESM.docx]

**Supplementary Table 3.** Primer sequences for RT-qPCR analysis; sgRNA cloning; clone ID and catalog numbers for shRNAs (Open Biosystems); Inhibitors; antibodies used-

| **Application** | **Gene symbol** | **Forward primer (5′-3′)** | **Reverse primer (5′-3′)** |
| --- | --- | --- | --- |
| RT-qPCR | HAT1 | tggcgatagaggcacaacag | acacgccggtaatcttccac |
|  | IGF1R | gtggagcccggcatcttact | cttggccccacggatatggt |
|  | ACTINB | gccgggacctgactgactac | tcttctccagggaggagctg |
|  |  |  |  |
| CRISPR | HAT1 sgRNA 1 Forward | caccgctacatgtcatgtcagccta |  |
|  | HAT1 sgRNA 1 Reverse | aaactaggctgacatgacatgtagc |  |
|  | HAT1sgRNA 2 Forward | caccggctacgctctttgcgaccgt |  |
|  | HAT1 sgRNA 2 Reverse | aaacacggtcgcaaagagcgtagcc |  |
|  | Non-targeting Forward | caccgaaaacaggacgatgtgcggcc |  |
|  | Non-targeting Reverse | aaacgccgcacatcgtcctgtttt |  |
|  |  |  |  |
|  | **Gene symbol** | **Clone ID** | **Catalog number** |
| shRNAs | HAT1 | TRCN0000034735 | RHS3979-9602143 |
|  | HAT1 | TRCN0000034737 | RHS3979-9602145 |
|  |  |  |  |
| **Antibodies** | **Protein** | **Source/catalog no.** | **Dilution** |
|  | HAT1 | Santa Cruz Biotechnology  (B-10: sc376268) | 1:200 |
|  | p-ERK1/2 | Cell signaling (4376) | 1:2000 |
|  | Total-ERK1/2 | Cell signaling (4695) | 1:2000 |
|  | p-IGF1R | Cell signaling (4568) | 1:2000 |
|  | Total-IGF1R | Cell signaling (3027) | 1:2000 |
|  | TGF-β | Cell signaling (3711) | 1:2000 |
|  | β-Catenin | Cell signaling (9562) | 1:2000 |
|  | ACTINB | Cell signaling (4970) | 1:2000 |
|  |  |  |  |
| **Immuno-fluorescence** | GM113 | BD Biosciences | 1:200 |
|  | HAT1 | Sigma Aldrich (Cat#HPA036788) | 1:500 |
|  | S100 | Biogenex Laboratories (Cat#AM058-5M) | 1:100 |
|  | HMB45 | Biogenex Laboratories (Cat#AM001-5M) | 1:100 |
|  | Alexaflour546 | Invitrogen (Cat# A-11030) | 1:100 |
|  | Cy5-Thyramide | Perkin Elmer (Cat#FP1117) | 1:50 |
|  |  |  |  |
| **Inhibitor** | **Concentrations** | **Source** |  |
| Vemurafenib | 0.1 μM, 1 μM and 2 μM | Selleckchem |  |
| Dabrafenib | 50 nM and 100 nM | Selleckchem |  |
| BMS-654807 | 0.1 and 1 μM | Selleckchem |  |
| SCH772984 | 0.2 and 1 μM | Selleckchem |  |
